# Fair innings? The utilitarian and prioritarian value of risk reduction over a whole lifetime

**DOI:** 10.1016/j.jhealeco.2020.102412

**Published:** 2021-01

**Authors:** Matthew D. Adler, Maddalena Ferranna, James K. Hammitt, Nicolas Treich

**Affiliations:** a Duke University, United States; b Harvard T.H. Chan School of Public Health, Harvard University, United States; c Harvard University, United States; d INRAE, Toulouse School of Economics, University Toulouse Capitole, France

**Keywords:** Social welfare function (SWF), Benefit-cost analysis (BCA), Value of statistical life (VSL), Fair innings, Social value of risk reduction (SVRR), Utilitarian, Prioritarian, Risk regulation

## Abstract

The social value of risk reduction (SVRR) is the marginal social value of reducing an individual’s fatality risk, as measured by some social welfare function (SWF). This Article investigates SVRR, using a lifetime utility model in which individuals are differentiated by age, lifetime income profile, and lifetime risk profile. We consider both the utilitarian SWF and a “prioritarian” SWF, which applies a strictly increasing and strictly concave transformation to individual utility.

We show that the prioritarian SVRR provides a rigorous basis in economic theory for the “fair innings” concept, proposed in the public health literature: as between an older individual and a similarly situated younger individual (one with the same income and risk profile), a risk reduction for the younger individual is accorded greater social weight even if the gains to expected lifetime utility are equal. The comparative statics of prioritarian and utilitarian SVRRs with respect to age, and to (past, present, and future) income and baseline survival probability, are significantly different from the conventional value per statistical life (VSL). Our empirical simulation based upon the U.S. population survival curve and income distribution shows that prioritarian SVRRs with a moderate degree of concavity in the transformation function conform to widely held views regarding lifesaving policies: the young should take priority but income should make no difference.

## 1 Introduction

Is it socially more important to save the lives of younger individuals, than to save the lives of the old? It seems hard to dispute that younger individuals should take priority with respect to lifesaving measures to the extent that age inversely correlates with life expectancy remaining, at least if the younger and older individuals are similarly situated with respect to the other determinants of well-being (health, income, etc.).^5^ If Anne is similarly situated to Bob, except for being younger, and a given reduction in Anne’s current mortality risk produces a larger increase in her life expectancy than the same reduction in Bob’s, the risk reduction for Anne seems socially more valuable.

But some have argued that the young should take priority with respect to lifesaving measures, and health policy more generally, on fairness grounds—not merely on the utilitarian basis that lifesaving measures directed at the young tend to yield a greater increase in life expectancy and expected lifetime well-being. Harris (1985, p. 91) introduced the idea of “fair innings” into the public health literature: “The fair innings argument requires that everyone be given an equal chance to have a fair innings, to reach the appropriate threshold but, having reached it, they have received their entitlement. The rest of their life is the sort of bonus which may be canceled when this is necessary to help others reach the threshold.” Others who have endorsed some version of the fair innings concept include Williams (1997); Daniels (1988); Lockwood (1988); Nord (2005); Bognar (2008, 2015); Ottersen (2013). The notion that the young should receive priority with respect to lifesaving measures is reflected, not merely in the academic literature on fair innings, but also in surveys of citizen preferences regarding health policy. (Bognar, 2008; Dolan et al., 2005; Dolan and Tsuchiya, 2012).

Bognar (2015, p. 254) uses the following thought experiment to crystallize the fair innings concept.

[Y]ou have only one drug and there are two patients who need it. The only difference between the two patients is their age. … You have to choose between saving: (C) a 20-year old patient who will live for 10 more years if she gets the drug; or (D) a 70-year old patient who will live for 10 more years if she gets the drug.

Both patients would spend the remaining ten years of their life in good health. So there is no difference in expected benefit. The only difference is how much they have already lived when they receive the benefit.

… [According to] the fairness-based argument for the fair innings view, you should … prefer C to D.

We’ll build on the suggestion of Bognar (2015) in using the term “fair innings” to mean the following: as between a policy that produces a given gain in expected lifetime well-being for a younger person, and an otherwise-identical policy that produces the same gain in expected lifetime well-being for an older person, it is ethically better for society to undertake the first policy.

While fair innings in this sense is an intuitively appealing idea, it is *not* supported by the current economic literature regarding the valuation of lifesaving. That literature generally focuses on benefit-cost analysis (BCA), which is the dominant tool in governmental practice for assessing fatality risk-reduction policies. The methodology of BCA does *not* support the idea that gains to the young are socially more valuable than equal gains for the old.^6^

In this Article, we examine the fair innings concept as part of a broader analysis of the use of social welfare functions (SWFs) to value risk reduction, and a comparison of the SWF framework to BCA. We show, in particular, that “prioritarian” SWFs place greater weight on gains to expected lifetime well-being accruing to younger rather than older individuals. We thus demonstrate that the fair innings concept has a rigorous basis in welfare economics—specifically in the SWF framework, not BCA.

BCA appraises government policies by summing individuals’ monetary equivalents—an individual’s monetary equivalent for a policy being the amount of money she is willing to pay or accept for it, relative to the status quo. In turn, the value per statistical life (VSL) is the concept that captures how BCA values fatality risk reduction. VSL is the marginal rate of substitution between an individual’s material resources (wealth, income, or consumption) and survival probability in a period. Put differently, VSL is the coefficient that translates a change in someone’s survival probability into a monetary equivalent. Individual *i*’s willingness to pay for a small improvement Δ*p_i_* in survival probability is approximately VSL*_i_* × Δ*p_i_*.

BCA, although now widespread, is controversial. A different framework for evaluating policy—one that has strong roots in economic theory and plays a major role in various bodies of scholarship within economics—is the social welfare function (SWF). The SWF framework measures policy impacts in terms of interpersonally comparable well-being, not monetary equivalents. Each possible outcome is a vector of individual well-being numbers, and a given policy is a probability distribution over such vectors. The SWF, abbreviated *W*(·), assigns a social value to a policy *P*, *W*(*P*), in light of the probability distribution over outcomes and, thus, well-being vectors that *P* corresponds to. On the SWF framework, see generally Adler (2012, 2019); Blackorby et al. (2005, chs. 2–4); Bossert and Weymark (2004); Weymark (2016).

In previous work (Adler et al. (2014)), we analyzed the application of the SWF framework to risk policies and compared how it values risk reduction to VSL. The key construct in our analysis was the social value of risk reduction (SVRR). The SVRR for individual *i* is the social value per unit of risk reduction to individual *i*, calculated for a marginal such reduction—social value as captured by the SWF *W*(·). SVRR*_i_* is just $\frac{\partial W}{\partial p_{i}}$, and the change in the SWF that occurs with a change Δ*p_i_* in individual *i*’s survival probability *p_i_* is approximately SVRR*_i_* × Δ*p_i_*.

Using the simple, one-period model that is often employed in the literature on VSL, Adler et al. (2014) calculated SVRR*_i_* for different types of SWFs: the utilitarian, “ex ante prioritarian,” and “ex post prioritarian” SWFs. (Utilitarianism ranks outcomes by summing well-being numbers, while prioritarianism does so by summing a strictly increasing and strictly concave transformation of well-being, thereby giving priority to those at lower well-being levels. The idea of utilitarianism dates back hundreds of years to the writings of Jeremy Bentham; prioritarianism is a more recent concept, pioneered by the moral philosopher Derek Parfit (2000). The ex ante and ex post prioritarian SWFs are two distinct specifications of prioritarianism for the case of uncertainty.) We analyzed the comparative statics of SVRR*_i_* and VSL*_i_* with respect to individual wealth and baseline risk.

The current Article significantly expands the analysis of Adler et al. (2014). We use a much richer model of individual resources and survival. An individual’s life has multiple periods, up to a maximum *T* (e.g., 100 years). Each individual is characterized by a lifetime risk profile (a probability of surviving to the end of each period, conditional on her being alive at its beginning); a lifetime income profile (an income amount which she earns in each period if she survives to its end); and a current age. This multi-period setup permits a more nuanced analysis of SVRR*_i_* and VSL*_i_*. In particular, we can now examine the comparative statics of SVRR*_i_* and VSL*_i_* with respect to an individual’s *age* as well as with respect to (past, present and future) income and baseline fatality risk.

The SWF framework is widely used in some areas of economics, such as optimal tax theory and climate economics. (Overviews of the use of the SWF framework in these two literatures are provided by Tuomala (2016) and Botzen and van den Bergh (2014) respectively.) It is also employed in health economics, with the SWF here typically being applied to a population characterized in terms of longevity and health states. (Bleichrodt et al., 2004; Dolan, 1998; Hougaard et al., 2013; Østerdal, 2005; Williams, 1997.) However, little research has been undertaken applying the SWF framework to the policy domain of fatality risk reduction—a major arena of governmental policymaking (Graham, 2008). We aim to make headway in exploring this important and understudied topic, and to raise its profile in the research community.

Section 2 sets forth the model and the SWFs we will consider. Section 3 analyzes the comparative statics of SVRR*_i_* and VSL*_i_* with respect to age. We provide a formal statement of the fair innings concept, via properties which we term “Priority for the Young” and “Ratio Priority for the Young.” We show that the ex ante prioritarian SVRR*_i_* and ex post prioritarian SVRR*_i_* both display Priority for the Young and indeed the logically stronger property of “Ratio Priority for the Young.” By contrast, VSL*_i_* does not have either property.^7^

Section 4 analyzes the comparative statics of SVRR*_i_* and VSL*_i_* with respect to income and baseline risk. Section 5 undertakes an empirical exercise, based on the U.S. population survival curve and income distribution, to illustrate the SVRR*_i_* concept and to estimate the impact of age and income on SVRR*_i_* and VSL*_i_*.

Our headline results are as follows. First, we demonstrate that the SWF framework—by contrast with BCA—provides a rigorous basis for the “fair innings” concept. The social value of risk reduction (SVRR), as calculated using an ex ante or ex post prioritarian SWF, gives extra social weight to risk reduction for younger individuals above and beyond the additional weight they receive in virtue of greater life expectancy remaining. (In an important article, Williams (1997) proposes to operationalize the “fair innings” concept via a non-utilitarian SWF applied to individuals’ quality-adjusted life expectancies; but Williams does not develop this proposal formally, as we do here.)

Second, we show that the manner in which BCA values risk reduction is significantly different from the SWF framework, *regardless* of which SWF is used (utilitarian, ex ante prioritarian, ex post prioritarian). These differences are multifold. The prioritarian SVRRs display Priority for the Young and Ratio Priority for the Young, while VSL does not. Further, as established in Section 4, the comparative statics of VSL with respect to income and baseline risk are different not only from the ex ante and ex post prioritarian SVRRs, but also from the utilitarian SVRR. Finally, Section 5 demonstrates that these analytic differences may be empirically quite significant. In particular, VSL increases much more steeply with income in each age group than the utilitarian SVRR, while the prioritarian SVRRs are flat or decrease with income.^8^

The text of the Article sets forth our analytic apparatus, defines relevant concepts, states our analytic results (as numbered propositions), and interprets these findings or explains the intuition behind them. However, so as to limit the length of the Article and increase readability, we do not include proofs of these propositions in the text. Instead, proofs are provided in an on-line Appendix.

This Article was drafted prior to the coronavirus pandemic of 2020. How to choose fatality-risk-reduction policies was an important topic before the pandemic, and will remain so after the pandemic abates. But the terrible events of 2020 underscore the significance of the questions we address here. One issue that quickly became salient as Covid-19 cases exploded was risk allocation. Which Covid-19 patients should take priority in receiving scarce medical equipment that would reduce the risk of dying from the disease, such as ventilators? Which uninfected individuals should go to the front of the line in receiving scarce protective equipment, such as N95 masks? The SWF framework provides a systematic methodology for answering such questions. It gives guidance in determining how the social value of reducing an individual’s fatality risk (in these cases, her risk of dying from Covid-19) should vary, or not, depending upon her age, income, and other characteristics. Understanding these relative social valuations, for three major SWFs—utilitarian, ex ante prioritarian, and ex post prioritarian—is precisely the topic of this Article.

## 2 Conceptual framework

### 2.1 Model of the population

There is a population of *N* individuals. The life of a given individual *i* is divided into periods 1, 2, …, *t*, …, *T*, with *T* the maximum number of periods that any individual can live. Each individual is characterized by an age, risk profile, and income profile, to be explained momentarily.

Calendar time is divided into the present time (also referred to as the “current” time), earlier times (“the past”), and later times (“the future”). This enables us to endow each individual *i* with an “age,” denoted as *A_i_*. We assume that individuals’ periods are synchronized, such that the present time is the *beginning* of some period for each of the *N* individuals. *A_i_* is the number of the present period for individual *i*. For example, if Betty has already lived 4 periods, and the present time is the beginning of period 5 of Betty’s life, then *A_Betty_* = 5, i.e., Betty’s “age” is 5.^9^ *A_i_* ≤ *T* and we also assume that *A_i_* ≥ 2.^10^

Death and survival are conceptualized as follows. Consider a given individual *i* and some period *t* in her life. Assuming the individual is alive at the beginning of period *t*, she may either die before the period ends, or survive to the end of the period (equivalently, be alive at the beginning of the following period). Let *p_i_*(*t*) denote individual *i*’s probability of surviving to the end of period *t*, conditional on being alive at the beginning of period *t*. We’ll generally refer to *p_i_*(*t*) as a “survival probability.” Individual *i* is characterized by a vector of such probabilities, one for each period up to *T*—for short, her “risk profile.”

In our model, these probabilities do not change as the individual ages. Individual *i* is endowed at birth with survival probabilities for each period *t* = 1, …, *T*; and *p_i_*(*t*) at the present time, the beginning of period *A_i_*, is this at-birth probability.^11^

Let π*_i_*(*t*; t*) denote individual *i*’s probability of surviving to the end of period *t* of her life, conditional on being alive at the beginning of period t*. In particular, π*_i_*(*t*; *A_i_*) is individual *i*’s probability of surviving to the end of period *t* of her life, conditional on being alive at the beginning of period *A_i_*—that is, her probability of surviving to the end of period *t*, conditional on her current age (*A_i_*). If *t* < *A_i_*, π*_i_*(*t*; *A_i_*) = 1. If *t* ≥ *A_i_*, $\pi_{i}(t;A_{i})={\prod_{s=A}^{t}}_{i}p_{i}(s)$.

Finally, let μ*_i_*(*t*; t*) denote individual *i*’s probability of living exactly *t* periods, conditional on being alive at the beginning of period t*. That is, μ*_i_*(*t*; t*) is the probability, conditional on being alive at the beginning of period t*, of surviving through the end of period *t* and then dying before the end of the next period. In particular, μ*_i_*(*t*; *A_i_*) is the individual’s probability of living exactly *t* periods, conditional on being alive at the beginning of period *A_i_*—conditional on her current age.

If *t* < (*A_i_* − 1), μ*_i_*(*t*; *A_i_*) = 0. If *t* = (*A_i_* −1), μ*_i_*(*t*; *A_i_*) is the individual’s probability, conditional on her current age, of not surviving the current period and instead living exactly (*A_i_* − 1) periods. That is μ*_i_*(*A_i_* − 1; *A_i_*) = 1 − *p_i_*(*A_i_*). Finally, if *t* ≥ *A_i_*, we have that: $\mu_{i}(t;A_{i})=\pi_{i}(t;A_{i})(1-p_{i}(t+1))$.

The earnings process is as follows: if an individual survives to the end of period *t*, she earns an income amount *y_i_*(*t*) > 0. Individual *i*, thus, is characterized by a vector of incomes, (*y_i_*(1), …, *y_i_*(*T*))—her “income profile.” An individual’s income profile, like her risk profile, is (in our model) given to the individual at birth and does not change as she ages.^12^

Period consumption, like period income, is modelled as occurring only if the individual survives to the end of the period. An individual’s consumption during period *t*, if she survives to the end of period *t*, is denoted *c_i_*(*t*). We assume “myopic” consumption: *c_i_*(*t*) = *y_i_*(*t*). The individual consumes in each period whatever she earns then, rather than saving earnings for future consumption or financing consumption by borrowing against future earnings.

“Myopic” consumption might occur because of imperfect markets—the individual lacks access to the financial instruments enabling her to save and borrow—or because of myopic thinking on the individual’s part. Given length constraints, we do not here analyze SVRR*_i_* with a multiperiod model *and* individual saving and borrowing. This is an important topic for future research.^13^

Individuals have a common lifetime utility function *U*(·), defined as the discounted sum of period utility. Let *u*(·) be the common period utility function and β = 1/(1 + *φ*), *φ* ≥ 0 the constant utility discount rate. *U_i_*(*s*) denotes the individual’s lifetime utility if she lives exactly *s* periods. $U_{i}(s)=\sum_{t=1}^{s}\beta^{t}u(y_{i}(t))$.^14^ We assume that *u*(·) is twice differentiable and that *u′*(·) > 0, *u′′*(·) < 0.

Note that the above formula for lifetime utility includes a term for a given period *t* iff^15^ the individual survives to the end of the period. If she doesn’t survive to the end of a given period, her period utility is zero. Further, our analysis presupposes that, if *i* does survive to the end of period *t*, with consumption *c_i_*(*t*) in that period, *u*(*c_i_*(*t*)) > 0. Note that if *u*(*c_i_*(*t*)) < 0, increasing *p_i_*(*t*) may have the effect of *lowering i*’s expected lifetime utility. We wish to focus here on the case in which risk reduction is beneficial to individuals—not the unusual case in which it may be harmful.^16^

We use *V_i_* to denote the expected lifetime utility of individual *i*, given his age, risk profile, and income profile. $V_{i}=\sum_{t=A_{i}-1}^{T}\mu_{i}(t;A_{i})U_{i}(t)$. This formula for *V_i_* is straightforward. Given that *i* is alive at the beginning of period *A_i_*, the possible lifespans for him are (*A_i_* −1), *A_i_*, …, *T*. The immediately preceding formula aggregates over these possible lifespans, calculating the lifetime utility for each possible lifespan and multiplying each possible lifetime utility *U_i_*(*t*) by its probability. A different formula for *V_i_*, useful in calculations, can also be derived (see Appendix). $V_{i}=\sum_{t=1}^{A_{i}-1}\beta^{t}u(y_{i}(t))+\sum_{t=A_{i}}^{T}\pi_{i}(t;A_{i})\beta^{t}u(y_{i}(t))$. This formula takes each period of *i*’s life, 1, …, *T*; calculates the discounted period utility for that period; multiplies by the probability of *i* surviving to the end of that period, conditional on his current age;^17^ and sums up over all the periods.

### 2.2 Social welfare functions (SWFs)

We’ll use the term “policy” to mean some course of action or inaction by the government. The status quo, therefore, is a “policy”: government chooses not to change individuals’ risk profiles or income profiles. A policy *intervention*, relative to the status quo, is also a “policy”: government changes individuals’ risk profiles and/or income profiles, specifically by changing present survival probabilities, future survival probabilities, present income amounts, and/or future income amounts. An individual’s risk profile or income profile with a given policy *P* is denoted with the superscript “*P*.” Thus $p_{i}^{P}(t)$ is *i*’s survival probability in period *t* with policy *P* and $y_{i}^{P}(t)$ is her period *t* income with policy *P*.

The SWF framework has three components: an interpersonally comparable well-being measure, which converts each possible outcome (a possible social consequence) into a vector of well-being numbers, one for each of the persons in the population; a rule for ranking well-being vectors; and an uncertainty module, namely a procedure for applying the rule to policies understood as probability distributions across outcomes. (Adler, 2012, 2019.) If individuals have a common utility function, then the well-being measure can be equated with that utility function (which is the approach we follow here). (Adler, 2019, ch. 3, app. D.) In what follows, we use “SWF” to mean the combination of a rule for ranking well-being vectors and an uncertainty module for that rule.

We consider three SWFs: the utilitarian SWF, the ex ante prioritarian SWF, and the ex post prioritarian SWF. Each assigns a score (a real number) to a given policy *P*, and ranks policies in the order of these scores. We’ll denote the utilitarian SWF as *W^U^*(·), the ex ante prioritarian SWF as *W^EAP^*(·), and the ex post prioritarian SWF as *W^EPP^*(·)—or, more compactly, as *W^U^*, *W^EAP^*, and *W^EPP^*. We’ll use *W*(·) as a generic term to indicate any SWF, with *W*(·) then specified as *W^U^*, *W^EAP^*, *W^EPP^*, or as some other SWF.^18^

The utilitarian rule ranks well-being vectors according to the sum of well-being. The standard procedure for applying the utilitarian rule under uncertainty is to sum individuals’ expected well-being. This yields the utilitarian SWF.

Definition 1a **The Utilitarian SWF**: $W^{U}(P)=\sum_{i=1}^{N}V_{i}^{P}$

The prioritarian rule ranks well-being vectors according to the sum of a strictly increasing and strictly concave transformation of individual well-being. Let *g*(·) denote some strictly increasing and strictly concave function. By summing *g*(·)-transformed well-being numbers, the prioritarian rule has the effect of giving greater weight to well-being changes affecting worse-off individuals. Assume that in well-being vector **w** a better-off individual is at well-being level *w_H_*, and a worse-off individual is at well-being level *w_L_*, with *w_H_* > *w_L_*. Let Δ*w* > 0 be a change in well-being. Well-being vector w* is identical to **w**, except that the better-off person is at well-being level *w_H_* + Δ*w*. Well-being vector w** is identical to **w**, except that the worse-off person is at well-being level *w_L_* + Δ*w*. The utilitarian rule is indifferent between w* and w**, while the prioritarian rule prefers w**, by virtue of the strict concavity of *g*(·). It prefers to give a fixed increment in well-being to a worse-off person rather than to a better-off one.

The two main approaches to applying the prioritarian rule under uncertainty are ex ante prioritarianism and ex post prioritarianism.^19^ (Adler, 2012, ch. 7; Adler, 2019, app. J; Adler et al., 2014.) Ex ante prioritarianism assigns a score to a given policy by calculating expected well-being for each individual; applying the transformation function, *g*(·), to each individual’s expected well-being; and then summing up these *g*(·)-transformed well-being expectations. Ex post prioritarianism assigns a score to a given policy by taking the expected value, for each individual, of her *g*(∙)-transformed well-being; and summing up these expected transformed well-being numbers.^20^ In a nutshell, the ex ante prioritarian formula is the sum across individuals of transformed expected well-being, while the ex post prioritarian formula is the sum across individuals of expected transformed well-being.

Ex ante and ex post prioritarianism each have a central place in the literature on prioritarianism because each has axiomatic advantages compared to the other. It can be shown that no procedure for applying the prioritarian rule under uncertainty can satisfy both the ex ante Pareto axioms, and a very plausible axiom of stochastic dominance. Ex ante prioritarianism satisfies the ex ante Pareto axioms, but violates stochastic dominance; ex post prioritarianism satisfies stochastic dominance, but violates the ex ante Pareto axioms.^21^ (Utilitarianism satisfies the ex ante Pareto axioms *and* stochastic dominance, but lacks the extra weighting for the worse off that is characteristic of prioritarianism, and that its proponents find to be ethically attractive.)

In the model here, the formulas for ex ante and ex post prioritarianism are as follows.

Definition 1b **The Ex Ante Prioritarian SWF**: $W^{EAP}(P)=\sum_{i=1}^{N}g(V_{i}^{P})$, with *g*(·) a strictly increasing and strictly concave function.

Definition 1c **The Ex Post Prioritarian SWF**: $W^{EPP}(P)=\sum_{i=1}^{N}\sum_{t=A_{i}-1}^{T}\mu_{i}^{P}(t;A_{i})g(U_{i}^{P}(t))$, with *g*(·) a strictly increasing and strictly concave function.

The utilitarian SWF is a specific SWF (a specific formula for ranking policies as a function of individuals’ ages, risk profiles, and income profiles) while the ex ante prioritarian SWF and ex post prioritarian SWF are, each, *families* of SWFs. The choice of a particular strictly increasing and strictly concave *g*(·) defines a specific *W^EAP^* and *W^EPP^*. Our analysis will be generic, holding true for any *g*(·). We do assume that *g*(·) is twice differentiable, so that *g*′(·) > 0 and *g*′′(·) < 0.^22^

Note that all three SWFs are defined in terms of individuals’ *lifetime* well-being. *W^U^* calculates each individual’s *expected lifetime well-being*, and sums across individuals. *W^EAP^* calculates each individual’s *transformed expected lifetime well-being*, and sums across individuals. *W^EPP^* calculates each individual’s *expected transformed lifetime well-being*, and sums across individuals. The application of SWFs to lifetime well-being has a strong ethical justification. (Adler, 2012, ch. 6). While much of the SWF literature uses one-period models for reasons of tractability, there is also a significant body of work using multiperiod or lifetime numbers as the input to an SWF.^23^ (For discussion of this literature, see Adler (2012, p. 245); Boadway (2012, pp. 86–106); Tuomala (2016, pp. 360–64).)

### 2.3 The social value of risk reduction (SVRR)

We’ll use the “O” superscript to denote an individual’s status quo income and risk profiles: *p_i_^O^*(*t*) is individual *i*’s status quo survival probability for period *t* and *y_i_*^O^(*t*) her status quo income for period *t*.

Assume that government enacts a policy intervention, relative to the status quo, at the beginning of the current period. Among other effects, the policy may change individual *i*’s current survival probability. Let Δ*p_i_* be this change: *i*’s current survival probability in the status quo is *p_i_^O^*(*A_i_*) and her current survival probability after the intervention is *p_i_^O^*(*A_i_*) + Δ*p_i_*.

We can now define SVRR*_i_*, which will be useful in understanding the impact of this policy intervention on social welfare.

Definition 2 **The Social Value of Risk Reduction (SVRR***_i_***)**: SVRR*_i_* for a given SWF *W*(·) is the partial derivative $\frac{\partial W}{\partial p_{i}(A_{i})}$ evaluated at *i*’s status quo risk and income profile.^24^

By the total differential approximation from calculus, the change in social welfare resulting from Δ*p_i_* is approximately SVRR*_i_* × Δ*p_i_*.^25^

Intuitively, SVRR*_i_* is the change in social welfare per unit of current risk reduction for individual *i*, as calculated for a marginal such reduction. To be sure, a governmental policy intervention may well have effects other than changing individuals’ current survival probabilities. It may also change their survival probabilities in future periods. And a risk-reduction intervention will surely have costs, which will be reflected in a change to individuals’ current and/or future incomes. The *total* effect of a policy intervention on social welfare will be approximately equal to the sum, across individuals, of SVRR*_i_* × Δ*p_i_* plus corresponding terms for changes to future survival probabilities and to incomes. SVRR*_i_* captures that *portion* of a policy intervention’s total impact on social welfare that results from the change to individual *i*’s current survival probability.

Further, by comparing SVRR*_i_* to SVRR*_j_*, for two individuals *i* and *j*—as we do below—we can determine the relative social impact of risk reductions for the two. Consider a change Δ*p* to someone’s current survival probability. That risk change, if accruing to individual *i*, results in a change of social welfare by approximately SVRR*_i_* × Δ*p*. If accruing to individual *j*, it results in a change of social welfare by approximately SVRR*_j_* × Δ*p*. Thus (for a small Δ*p*) the first social welfare change is larger than/smaller than/equal to the second iff SVRR*_i_* is larger than/smaller than/equal to SVRR*_j_*.

SVRR*_i_* is defined (Definition 2) as the partial derivative of the SWF with respect to individual *i*’s current survival probability, with this partial derivative evaluated at individual *i*’s status quo risk and income profiles. This reference to the status quo doesn’t limit the generality of the definition. For any assignment of income and risk profiles to individuals, we can take that assignment as the status quo and consider the social welfare impact of policy interventions relative to that baseline.

As a notational matter, we’ll also denote SVRR*_i_* for a generic SWF *W*(·) as *S_i_*; and SVRR*_i_* for *W^U^*, *W^EAP^*, and *W^EPP^* specifically as (respectively) $S_{i}^{U}$, $S_{i}^{EAP}$, and $S_{i}^{EPP}$.

Using the definition of SVRR*_i_* and of the SWFs (Definitions 1a, 1b, 1c), it is straightforward to calculate $S_{i}^{U}$, $S_{i}^{EAP}$, and $S_{i}^{EPP}$.^26^

Proposition 1a $S_{i}^{U}=-U_{i}^{O}(A_{i}-1)+\sum_{t=A_{i}}^{T}\frac{\mu_{i}^{O}(t;A_{i})}{p_{i}^{O}(A_{i})}U_{i}^{O}(t)$

Proposition 1b $S_{i}^{EAP}=g^{\prime }(V_{i}^{O})S_{i}^{U}$

Proposition 1c $S_{i}^{EPP}=-g(U_{i}^{O}(A_{i}-1))+\sum_{t=A_{i}}^{T}\frac{\mu_{i}^{O}(t;A_{i})}{p_{i}^{O}(A_{i})}g(U_{i}^{O}(t))$

We can provide intuitive explanations for these formulas, beginning with the utilitarian SVRR*_i_*. Observe that $S_{i}^{U}$ is equal to the difference between (1) individual *i*’s expected lifetime well-being conditional on surviving the current period, i.e., $\sum_{t=A_{i}}^{T}\frac{\mu_{i}^{O}(t;A_{i})}{p_{i}^{O}(A_{i})}U_{i}^{O}(t)$, and (2) her realized lifetime well-being if she dies during the current period (does not survive it), i.e., $U_{i}^{O}(A_{i}-1)$.

Consider the simple case in which individual *i* would die for certain during the current period, absent governmental intervention, and intervention ensures that she survives the period. In this case, clearly, the change in utilitarian social welfare that results from the intervention is the difference between individual *i*’s expected lifetime well-being conditional on surviving the current period, and her realized lifetime well-being if she dies during the current period. For short, let’s term this difference the “utilitarian gain from saving individual *i*.”

More generally, consider a policy which increases individual *i*’s current survival probability by Δ*p_i_*. The change in utilitarian social welfare that results from the Δ*p_i_* increase is just Δ*p_i_* multiplied by the utilitarian gain from saving individual *i*. Thus $S_{i}^{U}$, the marginal change in utilitarian social welfare per unit of current-period risk reduction for individual *i*, is nothing other than $-U_{i}^{O}(A_{i}-1)+\sum_{t=A_{i}}^{T}\frac{\mu_{i}^{O}(t;A_{i})}{p_{i}^{O}(A_{i})}U_{i}^{O}(t)$: the utilitarian gain from saving individual *i*.

The formula for the ex ante prioritarian SVRR*_i_*, $S_{i}^{EAP}$, is the utilitarian SVRR*_i_* multiplied by a weighting factor, $g^{\prime }(V_{i}^{O})$. This weighting factor is a function of the individual’s expected lifetime well-being, and decreases as expected lifetime well-being increases. It reflects the priority given by the ex ante prioritarian SWF to individuals at lower levels of expected lifetime well-being.

Finally, the formula for the ex post prioritarian SVRR*_i_*, $S_{i}^{EPP}$, is the same as that for the utilitarian SVRR*_i_*, except that transformed lifetime well-being, *g*(*U_i_*), is substituted for lifetime well-being *U_i_*. Consider the case in which individual *i* would die for certain during the current period, absent governmental intervention, and intervention ensures that she survives the period. In this case, the change in ex post prioritarian social welfare that results from the intervention is the difference between individual *i*’s expected transformed lifetime well-being conditional on surviving the current period, $\sum_{t=A_{i}}^{T}\frac{\mu_{i}^{O}(t;A_{i})}{p_{i}^{O}(A_{i})}g(U_{i}^{O}(t))$, and her realized transformed lifetime well-being if she dies during the current period, $g(U_{i}^{O}(A_{i}-1))$. For short, let’s term *this* difference the “ex post prioritarian gain from saving individual *i*.”

More generally, consider a policy which increases individual *i*’s current survival probability by Δ*p_i_*. The change in ex post prioritarian social welfare that results from the Δ*p_i_* increase is just Δ*p_i_* multiplied by the ex post prioritarian gain from saving individual *i*. Thus $S_{i}^{EPP}$, the marginal change in ex post prioritarian social welfare per unit of current-period risk reduction for individual *i*, is nothing other than the ex post prioritarian gain from saving individual *i*.

Note that our assumption that *u*(*y_i_*(*t*)) > 0 for all *i*, *t*—it is always better to survive a period than to die before its end—ensures that $S_{i}^{U}$, $S_{i}^{EAP}$, and $S_{i}^{EPP}$ > 0 for all *i*. Risk reduction is always a social benefit—whether social benefits are calculated using a utilitarian, ex ante prioritarian, or ex post prioritarian SWF.

It would be of interest to consider the relation between SVRR*_i_* as defined here and the partial derivative of the SWF with respect to *future* survival probability. Given space constraints, we do not address this topic, and instead focus in this Article on how the marginal social welfare impact of changes to *current* survival probability varies among individuals as a function of their ages, income profiles, and risk profiles.

### 2.4 Benefit-cost analysis (BCA) and the value of statistical life (VSL)

BCA is an evaluation methodology that assigns a score to each policy by summing up individuals’ monetary equivalents for that policy. (Adler, 2012, pp. 88–114; Boadway, 2016). In the model here, *ME_i_*(*P*), individual *i*’s monetary equivalent for policy *P*, is the change to her current status quo income that equalizes her expected utility as between the policy and the status quo. We use *B*(·) to denote the BCA methodology. *B*(*P*) is the score assigned by BCA to policy *P*: the sum of monetary equivalents for *P*.

Definition 3 **Benefit-Cost Analysis**: $B(P)=\sum_{i=1}^{N}ME_{i}(P)$, with *ME_i_*(*P*) as formally defined in the accompanying footnote.^27^

The value of statistical life (VSL) is standardly defined as the marginal rate of substitution between an individual’s material resources (wealth, income, or consumption) and survival probability. (Eeckhoudt and Hammitt, 2001; Evans and Smith, 2006; Kaplow, 2005; Hammitt, 2007.)

Consistent with this general approach, we define VSL*_i_* in our model as follows.

Definition 4 **The Value of Statistical Life (VSL***_i_***)**: $VSL_{i}=\frac{\partial V_{i}/\partial p_{i}(A_{i})}{\partial V_{i}/\partial y_{i}(A_{i})}$, with these partial derivatives evaluated at *i*’s status quo risk profile and income profile.^28^

The relation between VSL*_i_* and *B* is directly analogous to the relation between SVRR*_i_* and *W*. Just as $SVRR_{i}=\frac{\partial W}{\partial p_{i}(A_{i})}$, so $VSL_{i}=\frac{\partial B}{\partial p_{i}(A_{i})}$. This was true in the one-period model analyzed in Adler et al. (2014), and remains true in the lifetime model under consideration here.

Proposition 2a $VSL_{i}=\frac{\partial B}{\partial p_{i}(A_{i})}$, with $\frac{\partial B}{\partial p_{i}(A_{i})}$ evaluated at *i*’s status quo income and risk profiles.

Intuitively, VSL*_i_* is the marginal change in the sum of monetary equivalents per unit of current risk reduction for individual *i*, just as SVRR*_i_* is the marginal change in social welfare per unit of current risk reduction for individual *i*. Assume that a policy intervention changes individual *i*’s current survival probability by Δ*p_i_*. While the change in social welfare resulting from this risk change is approximately SVRR*_i_* × Δ*p_i_*, the change in the sum of monetary equivalents is approximately VSL*_i_* × Δ*p_i_*.^29^

From Definition 4, plus the formulas above for *V_i_* and the utilitarian SVRR*_i_*, it is straightforward to derive that *VSL_i_* equals the utilitarian SVRR*_i_* ($S_{i}^{U}$) divided by the expected marginal utility of *i*’s current income.

Proposition 2b $VSL_{i}=\frac{S_{i}^{U}}{p_{i}^{O}(A_{i})\beta^{A_{i}}u^{\prime }(y_{i}^{O}(A_{i}))}$

Given the formula for VSL*_i_* stated in Proposition 2b, it can be observed that the comparative statics of VSL*_i_* and the utilitarian SVRR*_i_* will be the same in the special case where all individuals in the status quo have the same expected marginal utility of current income. In that case, the denominator in this formula will be the same for all individuals, and VSL*_i_* then equals $S_{i}^{U}$ multiplied by a common positive constant. In general, however, relative to a generic status quo, VSL*_i_* and $S_{i}^{U}$ do not have the same comparative statics—as our analysis in Sections 3 and 4 below will demonstrate.

If we posit a perfect tax system that redistributes income so as to equalize individuals’ expected marginal utilities of (after-tax) present income, then the comparative statics of VSL*_i_* and $S_{i}^{U}$ will be the same. Observe, here, that equalizing incomes does not necessarily equalize expected marginal utilities of after-tax present income—since, for example, two individuals of the same age with the same after-tax present income but differing survival probabilities for the current period will have different expected marginal utilities.

A terminological note. We use the terms “SVRR*_i_*” and “VSL*_i_*” as the names for the concepts defined in Definitions 2 and 4 so as to emphasize that SVRR*_i_* and VSL*_i_* values are individual-specific. In general, given two distinct individuals *i* and *j*, it need not be the case that SVRR*_i_* = SVRR*_j_* and it need not be the case VSL*_i_* =VSL*_j_*. However, in what follows, so as to reduce clutter, we regularly drop the “*i*” subscript and use “SVRR” and “VSL” as shorthand, respectively, for “SVRR*_i_*” and “VSL*_i_*.”

## 3 Age effects and “priority for the young”

The effect of age on the SVRR has never been addressed by the academic literature. In this Section, we analyze what our model implies with respect to age effects on SVRR as well as VSL by considering two individuals *i* and *j*, with identical risk profiles and income profiles, but the first older than the second (*A_i_* > *A_j_*).

Both SVRR*_i_* and VSL*_i_* are determined by individual *i*’s status quo income profile and risk profile. (See Propositions 1a, 1b, 1c, 2b.) Thus, in analyzing the properties of SVRR*_i_* and VSL*_i_* in this Section as well as Section 4, we will not need to refer to incomes or probabilities, or to utilities as a function of incomes and probabilities, other than status quo values. We therefore remove the “*O*” superscript on incomes, probabilities, and utilities, which is implicit. *y_i_*(*t*) denotes $y_{i}^{O}(t)$, *p_i_*(*t*) denotes $p_{i}^{O}(t)$, *V_i_* denotes $V_{i}^{O}$, and so forth. Further, we often drop subscripts on incomes or probabilities where these are the same for *i* and *j*, e.g., *y*(*t*) indicates *y_i_*(*t*) =*y_j_*(*t*).

### 3.1 Age effects and the utilitarian SVRR

What are the relative magnitudes of $S_{i}^{U}$ and $S_{j}^{U}$, for two individuals of different ages (*A_i_* > *A_j_*) but with identical risk and income profiles? In other words, how does the utilitarian gain from saving an individual depend upon her age?

It can be shown that $S_{j}^{U}-S_{i}^{U}$ equals: $\sum_{t=A_{j}}^{A_{i}-1}\pi (t;A_{j}+1)\beta^{t}u(y(t))+\left(\pi (A_{i};A_{j}+1)-1\right)\sum_{t=A_{i}}^{T}\pi (t;A_{i}+1)\beta^{t}u(y(t))$. (See Appendix.)

Thus the utilitarian SVRR decreases/is unchanged/increases with age iff the value of this formula is positive/zero/negative.

The first term in this formula (for short, the “duration term”) is positive. By increasing the *younger* individual’s current survival probability, we increase her chance of surviving the periods *A_j_*, *A_j_* +1, …, *A_i_* – 1 in her life, and that probability change for each such period yields an increment in expected lifetime well-being (by increasing her chance of accruing consumption utility with respect to that period). This increment to expected lifetime well-being with respect to periods *A_j_, A_j_* +1, …, *A_i_* − 1 does not occur if we increase the *older* individual’s survival probability, since he has already survived those periods.

The second term in the formula above (for short, the “risk term”) is negative. By increasing either individual’s current survival probability, we increase that individual’s chance of surviving periods *A_i_*, *A_i_* + 1, …, *T* in his or her life, and thereby increase his or her chance of accruing consumption utility with respect to those periods. The risk term captures the *difference* between the magnitude of this benefit for the younger individual and its magnitude for the older one. Since the older individual is sure to be alive at the beginning of period *A_i_*, while the younger individual is not, this difference is negative.

Clearly, if income can increase with age, the magnitude of the risk term may exceed that of the duration term, and thus the utilitarian gain from saving the older individual may be greater than that of saving the younger one. What if constant income is assumed? With a constant income profile and a constant risk profile, the duration term predominates and the utilitarian SVRR decreases with age. More generally, it can be shown that if the income and risk profiles are such that income does not increase with age and survival probabilities do not increase with age, then the utilitarian SVRR decreases with age. (See Appendix.)

### 3.2 Age effects and the ex ante prioritarian SVRR

A simple manipulation shows that $S_{j}^{EAP}-S_{i}^{EAP}=g^{\prime }(V_{j})\left(S_{j}^{U}-S_{i}^{U}\right)+S_{i}^{U}\left(g^{\prime }(V_{j})-g^{\prime }(V_{i})\right)$. We noted immediately above in discussing the utilitarian SVRR that the quantity $\left(S_{j}^{U}-S_{i}^{U}\right)$ equals a positive “duration” term plus a negative “risk” term. The first part of the formula here, namely $g^{\prime }(V_{j})\left(S_{j}^{U}-S_{i}^{U}\right)$, incorporates those terms. This part is positive iff $\left(S_{j}^{U}-S_{i}^{U}\right)$ is positive. The second part of the formula here, $S_{i}^{U}\left(g^{\prime }(V_{j})-g^{\prime }(V_{i})\right)$, is a third term (“priority for the young”), which is always positive. Because *V_i_* > *V_j_* (the older individual has greater expected lifetime well-being) and *g*(·) is strictly concave, *g*′(*V_i_*) < *g*′(*V_j_*).

The intuition behind the formula is as follows. Ex ante prioritarian social welfare, *W^EAP^*, is the sum of individuals’ transformed expected lifetime well-beings— transformed by a strictly increasing and strictly concave *g*(·) function. The effect of this transformation is to give greater social weight to changes in expected lifetime well-being that accrue to individuals at lower levels of expected lifetime well-being. The differential ex-ante-prioritarian benefit of saving a younger rather than older individual reflects the differential gains to expected lifetime well-being of saving the younger one $\left(S_{j}^{U}-S_{i}^{U}\right)$. But it also reflects the fact that the younger individual has a lower level of expected lifetime well-being and thus takes priority ($g^{\prime }(V_{j})>g^{\prime }(V_{i})$).

We now define “Priority for the Young” more formally.

Definition 5 **Priority for the Young**: Consider any two individuals *i* and *j* with identical risk profiles and income profiles (*p_i_*(*t*) = *p_j_*(*t*) and *y_i_*(*t*) = *y_j_*(*t*) for all *t*), and such that *A_i_* > *A_j_*. SVRR for a given SWF *W*(·) displays “Priority for the Young” iff the following is true for any such *i* and *j*: $S_{j}^{U}-S_{i}^{U}\geq 0\Rightarrow S_{j}-S_{i}>0$. Similarly, VSL displays Priority for the Young iff the following is true for any such *i* and *j*: $S_{j}^{U}-S_{i}^{U}\geq 0\Rightarrow VSL_{j}-VSL_{i}>0$.

Priority for the Young is a precise expression, using the SVRR formalism, of the fair innings concept. Recall our informal definition of “fair innings” in the Introduction (Section 1): As between a policy that produces a given gain in expected lifetime well-being for a younger person, and an otherwise-identical policy that produces the same gain in expected lifetime well-being for an older person, it is ethically better for society to undertake the first policy. Recall too (Section 3.1) that the utilitarian SVRR*_i_*, $S_{i}^{U}$, is equal to the per-unit gain to expected lifetime well-being from reducing *i*’s current fatality risk. If *i*’s current survival probability increases by Δ*p_i_*, his expected lifetime well-being increases by $\Delta p_{i}\times S_{i}^{U}$.

If SVRR*_i_* for a given SWF *W*(·) displays Priority for the Young, then it never assigns a smaller or equal value to risk reduction for the younger individual if the utilitarian risk-reduction value is larger for the younger than for the older individual. (If the SWF displays Priority for the Young, it follows that: $S_{j}^{U}-S_{i}^{U}>0\Rightarrow S_{j}-S_{i}>0$.) Further, if the utilitarian risk-reduction values are equal, the SWF places a larger value on risk reduction for the younger individual. (If the SWF displays Priority for the Young, it also follows that: $S_{j}^{U}-S_{i}^{U}=0\Rightarrow S_{j}-S_{i}>0$.).

Proposition 3a The Ex Ante Prioritarian SVRR displays Priority for the Young.

We can illustrate why ex ante prioritarianism satisfies Priority for the Young using the Bognar (2015) thought experiment presented in the Introduction. Consider two patients, a younger patient *j* and an older patient *i*, who are respectively at the beginning of periods two and three of their lives. The maximum lifespan is three periods. The patients have a common risk profile, with *p*_2_ the common survival probability for period two and *p*_3_ the common survival probability for period three. Assume also that the patients are equally well off, materially. Each faces the same, constant, income profile, with period utility normalized to 1 and a zero utility discount rate.

Finally, assume that *p*_3_ is close to zero, so that $S_{j}^{U}\approx S_{i}^{U}=1$ (as shown in note 30). Thus, if we have one dose of a drug that can increase a patient’s current survival probability by some fixed increment, utilitarianism is indifferent between giving the drug to the younger or the older patient; the utilitarian SVRRs are approximately equal. However, we easily obtain that $S_{j}^{EAP}\approx {g^{\prime }}^{}(1+p_{2})>S_{i}^{EAP}\approx g^{\prime }(2)$, by the concavity of *g*(·).^30^ Ex ante prioritarianism tells us to give the drug to the younger individual, who has a lower expected lifetime well-being (it is uncertain whether she will survive the second period, while the older patient will definitely live at least two periods). Ex ante prioritarianism gives greater weight to a given increase in expected lifetime well-being if it accrues to an individual at a lower level of expected lifetime well-being, and so the younger patient takes priority.

Not only does ex ante prioritarianism satisfy Priority for the Young. We can prove a logically stronger result, namely that the relative social value of risk reduction for young versus old individuals is always greater with ex ante prioritarianism than with utilitarianism. $\left(S_{j}^{EAP}/S_{i}^{EAP})>(S_{j}^{U}/S_{i}^{U}\right)$. If utilitarianism prefers to reduce the younger individual’s risk (the utilitarian gain from saving her is greater), ex ante prioritarianism has a yet greater degree of priority for the young. If utilitarianism is indifferent (the utilitarian gains are equal), ex ante prioritarianism gives priority to the young. Finally, although ex ante prioritarianism may prefer to reduce the risk of the older individual (if the utilitarian gain from saving her is sufficiently greater), in this case it always gives less priority to the older individual than utilitarianism does.

Definition 6 **Ratio Priority for the Young**: Consider any two individuals *i* and *j* with identical risk profiles and income profiles (*p_i_*(*t*) = *p_j_*(*t*) and *y_i_*(*t*) = *y_j_*(*t*) for all *t*), and such that *A_i_* > *A_j_*. SVRR for a given SWF *W*(·) displays “Ratio Priority for the Young” iff the following is true for any such *i* and *j*: $\left(S_{j}/S_{i})>(S_{j}^{U}/S_{i}^{U}\right)$. Similarly, VSL displays Ratio Priority for the Young iff the following is true for any such *i* and *j*: $\left(VSL_{j}/VSL_{i})>(S_{j}^{U}/S_{i}^{U}\right)$.

Proposition 3b The Ex Ante Prioritarian SVRR displays Ratio Priority for the Young.

Note that Ratio Priority for the Young is a logically stronger property than Priority for the Young. If SVRR for a given *W*(·) satisfies Ratio Priority for the Young, then necessarily it satisfies Priority for the Young; but the converse is not true.^31^

Both Priority for the Young and Ratio Priority for the Young are defined relative to a utilitarian baseline. It is an immediate logical consequence of these definitions that the utilitarian SVRR displays neither property. This is not a mathematical result, but simply the logical upshot of our definitions, and so we don’t include the utilitarian SVRR in the numbered propositions concerning Priority for the Young and Ratio Priority for the Young.

### 3.3 Age effects and the ex post prioritarian SVRR

It can be shown that $S_{j}^{EPP}-S_{i}^{EPP}$ equals: $\sum_{t=A_{j}}^{A_{i}-1}\mu (t;A_{j}+1)g(U(t))+(\pi (A_{i};A_{j}+1)-1)\sum_{t=A_{i}}^{T}\mu (t;A_{i}+1)g(U(t))+\left(g(U(A_{i}-1))-g(U(A_{j}-1))\right)$

Although this formula is different from $S_{j}^{EAP}-S_{i}^{EAP}$, it nonetheless reflects the same three factors. The first term of the formula is a positive “duration term,” reflecting the increased chance for the younger individual of surviving periods *A_j_* through *A_i_* − 1; the second term is a negative “risk term,” reflecting the chance she will not survive to period *A_i_*; and the third term is a positive “priority for the young” term.

We saw above that the ex ante prioritarian SVRR displays “Priority for the Young”: it prefers to reduce the younger individual’s risk even when utilitarianism is indifferent, and prefers to do so whenever utilitarianism does. The same is true for the ex post prioritarian SWF.

Proposition 3c The Ex Post Prioritarian SVRR displays Priority for the Young.

The intuition for this result is as follows. As explained earlier, the ex post prioritarian SVRR, $S_{i}^{EPP}=-g(U_{i}(A_{i}-1))+\sum_{t=A_{i}}^{T}\frac{\mu_{i}(t;A_{i})}{p_{i}(A_{i})}g(U_{i}(t))$, is the difference between individual *i*’s expected transformed lifetime well-being conditional on surviving the current period, and her transformed lifetime well-being if she does not survive. Equivalently, it is the *expected difference* between her transformed lifetime well-being conditional on surviving the current period (given her possible lifespans if she does survive and their probabilities), and her transformed lifetime well-being if she does not survive.

Consider now two individuals, one (*j*) younger than the second (*i*), with a common risk and income profile. The ex post prioritarian SWF places less value on a risk reduction for *i* than for *j* because *i*’s lifetime well-being if she dies during the current period, *U*(*A_i_* − 1), is greater than *j*’s if she dies during the current period, *U*(*A_j_* − 1)—and thus the very same increase in lifetime well-being for the two individuals translates into a smaller change in transformed lifetime well-being for *i*. Assume that *i*, if she survives the period, has probability δ of realizing a level of lifetime well-being which is Δ*U* greater than her level of lifetime well-being if she dies now. And assume that the same is true for *j*. The utilitarian value of a chance δ of increment Δ*U* is the same for both individuals, namely δ(Δ*U*). The ex post prioritarian value of a chance δ for individual *j* of increment Δ*U* is $\delta \left(g(U(A_{j}-1)+\Delta U)-g(U(A_{j}-1))\right)$, while for *i* it is $\delta \left(g(U(A_{i}-1)+\Delta U)-g(U(A_{i}-1))\right)$. The first value is greater than the second by virtue of the strict concavity of *g*(·), because *U*(*A_j_* − 1) < *U*(*A_i_* − 1).

We can again use the Bognar (2015) thought experiment, now to illustrate why ex post prioritarianism satisfies Priority for the Young. Following the example in Section 3.2 above, we have that utilitarianism is (approximately) indifferent between giving the drug to the younger patient and giving it to the older one, if *p*_3_ is small. $S_{j}^{U}\approx S_{i}^{U}=1.$ However, $S_{i}^{EPP}=g(3)-g(2)$, while $S_{j}^{EPP}\approx g(2)-g(1)$ if *p*_3_ is small.^32^ (If the older individual survives the period, her expected transformed lifetime well-being is *g*(3); her transformed lifetime well-being if she does not is *g*(2). If the younger individual survives the period, her expected transformed lifetime well-being, with *p*_3_ small, is approximately *g*(2); her transformed lifetime well-being if she does not is *g*(1).) By the concavity of *g*(·), *g*(3) − *g*(2) < *g*(2) − *g*(1).

We saw above that ex ante prioritarianism satisfies not merely Priority for the Young but also the (logically stronger) Ratio Priority for the Young. The same is true for ex post prioritarianism.

Proposition 3d The Ex Post Prioritarian SVRR Displays Ratio Priority for the Young

### 3.4 Age effects and VSL

As is well known, the effect of age on VSL is ambiguous (Aldy and Viscusi, 2007; Hammitt, 2007). In our model, age impacts VSL via its effect on the utilitarian SVRR (the numerator of VSL), plus an additional effect: the change in expected marginal utility of consumption (the denominator of VSL) with age.

As throughout this Section, let *i* and *j* be two individuals with identical risk and income profiles, and such that *A_i_* > *A_j_*. Let $C_{i}=p(A_{i})\beta^{A_{i}}u^{\prime }(y(A_{i}))$ and similarly for *C_j_*. Then VSL*_j_* − VSL*_i_* equals:

$$\frac{1}{C_{j}}\left(S_{j}^{U}-S_{i}^{U}\right)+S_{i}^{U}\left(\frac{1}{C_{j}}-\frac{1}{C_{i}}\right)$$

Note that the expected marginal utility of consumption for the younger individual (*C_j_*) may be larger than for the older individual (*C_i_*)—which can occur if the younger individual has less consumption and/or a greater current survival probability. If *C_j_* > *C_i_*, the second term in the above formula for VSL*_j_* − VSL*_i_* will be negative even if $S_{j}^{U}=S_{i}^{U}$. Further, if $S_{j}^{U}>S_{i}^{U}$, the second term will again be negative if *C_j_* > *C_i_*, and its magnitude may exceed that of the first term.

In short, it is not necessarily the case that $S_{j}^{U}-S_{i}^{U}=0\Rightarrow VSL_{j}-VSL_{i}>0$; and it is not necessarily the case that $S_{j}^{U}-S_{i}^{U}>0\Rightarrow VSL_{j}-VSL_{i}>0$.

Proposition 3e VSL does not display Priority for the Young.

In other words: BCA may prefer a risk reduction for the older individual even if the utilitarian gains are equal, indeed even if the utilitarian gain from saving the younger one is larger.

Because Ratio Priority for the Young is a logically stronger property than Priority for the Young^33^ —if VSL were to display the former, it would necessarily display the latter—the proposition that VSL fails to display Priority for the Young implies (by contraposition) that it fails to display Ratio Priority for the Young.

Proposition 3f VSL does not display Ratio Priority for the Young.

### 3.5 Age effects: a summary

Table 1 summarizes the results of our analysis of age effects on the utilitarian, ex ante prioritarian, and ex post prioritarian social values of risk reduction (SVRR), and on VSL.

**Table 1:** SVRRs, VSL, and Priority for the Young.

	Priority for the Young	Ratio Priority for the Young
Utilitarian SVRR*	---	---
Ex Ante Prioritarian SVRR	Yes	Yes
Ex Post Prioritarian SVRR	Yes	Yes
VSL	No	No

* Because Priority for the Young and Ratio Priority for the Young are defined as a stronger preference for the young than the utilitarian preference, it is true by definition that the utilitarian SVRR doesn’t have these properties—and so these cells in the table are left blank.

One “takeaway” from our analysis is that the concept of prioritarianism, in both its ex ante and ex post variants, provides a rigorous basis for the fair innings concept—as precisely expressed by the properties Priority for the Young and Ratio Priority for the Young. Ex ante prioritarian social welfare, *W^EAP^*, is the sum of a strictly increasing and strictly concave transformation function, *g*(·), applied to each individual’s expected lifetime well-being. The ex ante prioritarian SVRR has the priority-for-the-young properties because a given increment in expected lifetime well-being is accorded greater social weight when provided to an individual at a lower level of expected lifetime well-being. The ex post prioritarian SVRR has the priority-for-the-young properties for a different reason. The ex post prioritarian SWF, *W^EPP^*, applies the *g*(·) function to individuals’ possible *realized* (not expected) lifetime well-being levels; calculates expected transformed lifetime well-being for each individual; and sums across individuals. As compared to older persons with the same risk and income profile, younger persons face a lottery over possible realized lifetime well-being levels with a greater chance of lower realized levels, and a smaller chance of higher realized levels. A given increment in realized lifetime well-being is accorded greater social weight by *W^EPP^*, if provided to someone at a lower level of realized lifetime well-being.

For those familiar with the literature on prioritarianism under uncertainty, it will be striking that *both* ex ante prioritarianism *and* ex post prioritarianism display Priority for the Young and Ratio Priority for the Young. This literature demonstrates a range of significant axiomatic differences between the two variants of prioritarianism (including, as mentioned above, with respect to the ex ante Pareto and stochastic dominance axioms). (Adler, 2012 ch. 7; Adler, 2019 app. J). The current analysis shows that, notwithstanding these important differences, the two approaches are alike in supporting the fair innings concept.

Our analysis also extends an important finding of Adler et al. (2014). That article, as mentioned, used a single-period model which was not suited to study age effects. What it *did* study was the effect of income and baseline risk on the utilitarian, ex ante prioritarian, and ex post prioritarian SVRRs and on VSL. Here, Adler et al. (2014) found that BCA and the SWF framework value risk reduction in significantly different ways. The present analysis confirms that finding, now with respect to age effects. By contrast with ex ante and ex post prioritarian SVRRs, VSL does not display Priority for the Young or Ratio Priority for the Young.

## 4 The effects of income and baseline risk

We now consider how SVRR and VSL vary between individuals of the same age, but with different income or risk profiles.

### 4.1 Sensitivity to income

We consider first whether SVRR and VSL increase, decrease, or are unchanged by a *single-period increment in income*. Two individuals *i* and *j* are identical in age (*A_i_* = *A_j_*), in their risk profiles, and in their income profiles except that *y_j_*(*t*) = *y_i_*(*t*) + Δ*y*, Δ*y* > 0, for some single period *t*. The period in which the individuals’ incomes differ can be the current period, in which case *t* =*A_i_* = *A_j_*, or it can be a past or future period. We determine whether SVRR*_j_* > SVRR*_i_*, SVRR*_j_* = SVRR*_i_*, or SVRR*_j_* < SVRR*_i_* by examining the sign of $\frac{\partial S_{i}}{\partial y_{i}(t)}$. We proceed analogously for VSL.

We find as follows.

Proposition 4a The utilitarian SVRR is unchanged by a single-period increment to past income. It increases with a single-period increment to present or future income.

Proposition 4b The ex ante prioritarian SVRR decreases with a single-period increment to past income. The effect of a single-period increment to present income or future income on the ex ante prioritarian SVRR is ambiguous.^34^

Proposition 4c The ex post prioritarian SVRR decreases with a single-period increment to past income. It increases with a single-period increment to present or future income.

Proposition 4d VSL is unchanged by a single-period increment to past income. It increases with a single-period increment to present or future income.

Although we do not prove the propositions here (see Appendix), the following remarks may help to explain them. *Utilitarian SVRR*. The utilitarian SVRR is “history independent.” As shown in the Appendix, the formula for $S_{i}^{U}$ can be restated so as to make evident that it does not depend upon individual *i*’s past survival probabilities or incomes. In particular, then, if *i* and *j* are identical except that *j* has a higher income than *i* in a single past period, $S_{i}^{U}$ = $S_{j}^{U}$. The utilitarian SVRR increases with a single-period increment to present or future income because preventing the current death of an individual with greater present or future income produces a larger gain in expected lifetime well-being.

*Ex Ante Prioritarian SVRR.* Unlike the utilitarian SVRR, the ex ante prioritarian SVRR is “history dependent.” While the formula for $S_{i}^{EAP}$ does not depend upon *i*’s past survival probabilities, it *does* take account of her past income. The explanation for why the ex ante prioritarian SVRR decreases with a single-period increment to past income is the following: If individuals *i* and *j* are identical except that *j* has greater past income, then preventing either of their deaths in the current period produces the same increment in expected lifetime well-being, but individual *i* has a lower baseline level of expected lifetime well-being, thus takes priority under *W^EAP^*.

Why does a single-period increment to present or future income have an ambiguous effect on the ex ante prioritarian SVRR? In a nutshell, the reason is this: If the two individuals are identical except that *j* has greater present or future income than *i*, then *i* has a lower baseline level of expected lifetime well-being, so takes priority under *W^EAP^*; but reducing her current risk produces a smaller increase in expected lifetime well-being than reducing *j*’s current risk. Whether *W^EAP^* prefers to reduce individual *i*’s current risk or instead individual *j*’s depends upon the concavity of the transformation function *g*(·). In particular, we show that if *g*(·) is such that the coefficient of relative risk aversion is always less than or equal to 1, a single-period increment to present or future income will increase the ex ante prioritarian SVRR.

*Ex Post Prioritarian SVRR.* The ex post prioritarian SVRR, too, is history dependent. The formula for $S_{i}^{EPP}$, like the formula for $S_{i}^{EAP}$, does not take account of *i*’s past survival probabilities but does take account of her past incomes. Further, like the ex ante prioritarian SVRR, the ex post prioritarian SVRR decreases with a single-period increment to past income. However, unlike its ex ante counterpart, the ex post prioritarian SVRR *always* increases with a single-period increment to present or future income.

The reason for the divergence between $S_{i}^{EAP}$ and $S_{i}^{EPP}$ as regards sensitivity to present or future income is subtle. The social value, as per *W^EPP^*, of preventing an individual from dying during the current period is the expected difference between the transformed lifetime well-being of the longer lives she might lead were she to survive the current period, and the transformed lifetime well-being of her life were it to end now. Increasing present or future income *increases* that expected difference in transformed lifetime well-being.

*VSL.* Because VSL*_i_* equals $S_{i}^{U}$ divided by the expected marginal utility of *i*’s current income, the comparative statics of VSL with respect to past and future income are the same as for the utilitarian SVRR. Further, because the utilitarian SVRR (the numerator of VSL) is increasing in current income, and the denominator is decreasing, VSL also increases in current income—indeed more quickly than the utilitarian SVRR.

Next, we consider the effect on SVRR and VSL of an increment to *permanent income*. Two individuals *i* and *j* are identical except that *y_j_*(*t*) = *y_i_*(*t*) + Δ*y*, Δ*y* > 0, for every period *t* = 1 to *T*. We find as follows.

Proposition 5a The utilitarian SVRR increases with an increment to permanent income.

Proposition 5b The effect of an increment to permanent income on the ex ante prioritarian SVRR is ambiguous.

Proposition 5c The effect of an increment to permanent income on the ex post prioritarian SVRR is ambiguous.

Proposition 5d VSL increases with an increment to permanent income.

### 4.2 Sensitivity to baseline risk

We consider first whether SVRR and VSL increase, decrease, or are unchanged by a *single-period increment in survival probability*. Two individuals *i* and *j* are identical except that *p_j_*(*t*) = *p_i_*(*t*) + Δ*q*, Δ*q* > 0, for some single period *t*. We determine whether SVRR*_j_* > SVRR*_i_*, SVRR*_j_* = SVRR*_i_*, or SVRR*_j_* < SVRR*_i_* by examining the sign of $\frac{\partial S_{i}}{\partial p_{i}(t)}$. We proceed analogously for VSL.

None of the SVRRs, nor VSL, take account of *past* survival probabilities. (The ex ante prioritarian and ex post prioritarian SVRRs are history-dependent because they take account of past incomes; but their formulas do not also depend upon past survival probabilities.) We therefore focus on the case of a one-period change to present survival probability (*t* = *A_i_* = *A_j_*) or future survival probability.

Proposition 6a The utilitarian SVRR is unchanged by a single-period increment to present survival probability. It increases with a single-period increment to future survival probability.

Proposition 6b The ex ante prioritarian SVRR decreases with a single-period increment to present survival probability. The effect of a single-period increment to future survival probability on the ex ante prioritarian SVRR is ambiguous.

Proposition 6c The ex post prioritarian SVRR is unchanged by a single-period increment to present survival probability. It increases with a single-period increment to future survival probability.

Proposition 6d VSL decreases with a single-period increment to present survival probability. It increases with a single-period increment to future survival probability.

Again, see Appendix for proofs of the results. The following remarks may help to explain them.

*Utilitarian SVRR*. The formula for $S_{i}^{U}$ can be rewritten so that only future survival probabilities, not the current survival probability *p_i_*(*A_i_*), show up in the formula. An increment to current survival probability therefore has no effect on the utilitarian SVRR. The utilitarian SVRR is increasing with a one-period increment to future survival probability because preventing a current death produces a bigger increase in expected lifetime well-being if the individual has a lower chance of dying in future periods.

*Ex Ante Prioritarian SVRR.* The ex ante prioritarian SVRR is decreasing in current survival probability: An individual with lower present survival probability has a lower level of expected lifetime well-being, hence takes priority under *W^EAP^*.

An individual with lower future survival probability also has a lower level of expected lifetime well-being, hence also takes priority under *W^EAP^*, but reducing her current risk produces a smaller increase in expected lifetime well-being. Which effect predominates depends upon the concavity of *g*(·). Hence the impact on the ex ante prioritarian SVRR of a single-period increment to future survival probability is ambiguous. We demonstrate, specifically, that if *g*(·) is such that the coefficient of relative risk aversion is always less than or equal to 1, a single-period increment to future survival probability will increase the ex ante prioritarian SVRR.

*Ex Post Prioritarian SVRR*. The ex post prioritarian SVRR is unchanged by an increment to current survival probability. (The formula for $S_{i}^{EPP}$ can be rewritten so that *p_i_*(*A_i_*) drops out of that formula.) It is increasing with a single-period increment to future survival probability. As noted earlier, the social value, as per *W^EPP^*, of preventing an individual from dying during the current period is the expected difference between the transformed lifetime well-being of the longer lives she might lead were she to survive the current period, and the transformed lifetime well-being of her life were it to end now. Increasing future survival probability *increases* that expected difference in transformed lifetime well-being.

*VSL.* VSL is the utilitarian SVRR divided by a denominator that increases with current survival probability, and is independent of future survival probability. Because the utilitarian SVRR is unchanged by a single-period increment to current survival probability, VSL decreases with such an increment. Because the utilitarian SVRR increases with a single-period increment to future survival probability, VSL also increases with such an increment.

Next, we consider the effect on SVRR and VSL of a *permanent* increment to survival probability. Two individuals *i* and *j* are identical except that *p_j_*(*t*) = *p_i_*(*t*) + Δ*q*, Δ*q* > 0, for every present and future period *t* (for every *t* ≥ *A_i_* = *A_j_*). We find as follows.

Proposition 7a The utilitarian SVRR increases with a permanent increment to survival probability.

Proposition 7b The effect of a permanent increment to survival probability on the ex ante prioritarian SVRR is ambiguous.

Proposition 7c The ex post prioritarian SVRR increases with a permanent increment to survival probability.

Proposition 7d The effect of a permanent increment to survival probability on VSL is ambiguous.

### 4.3 Income and baseline risk: summary

The comparative statics of the SVRRs and VSL with respect to income and survival probability are summarized in Table 2.

**Table 2:** Comparative Statics of SVRRs and VSL with respect to Income and Survival Probability.

	Income: Single-period difference	Income: permanent difference	Survival probability: single-period difference	Survival probability: permanent difference
**Utilitarian SVRR**	Past period: *Unchanged* Current period: *Increasing* Future period: *Increasing*	*Increasing*	Current period: *Unchanged* Future period: *Increasing*	*Increasing*
**Ex Ante Prioritarian SVRR**	Past period: *Decreasing* Current period: Ambiguous* Future period: Ambiguous*	*Ambiguous*	Current period: *Decreasing* Future period: Ambiguous*	*Ambiguous*
**Ex Post Prioritarian SVRR**	Past period: *Decreasing* Current period: *Increasing* Future period: *Increasing*	*Ambiguous*	Current period: *Unchanged* Future period: *Increasing*	*Increasing*
**VSL**	Past period: *Unchanged* Current period: *Increasing* Future period: *Increasing*	*Increasing*	Current period: *Decreasing* Future period: *Increasing*	*Ambiguous*

* The ex ante prioritarian SVRR increases with a single-period increment to current or future income, and increases with a single-period increment to future survival probability, if g(·) is such that its coefficient of relative risk aversion is always less than or equal to 1.

Much about this table is noteworthy. First, timing matters. Whether individuals who differ with respect to income, or with respect to survival probability, have divergent SVRRs or VSL depends upon whether the income or survival probability difference occurs in the past, the present, or the future. Consider the columns for “income: single-period difference” and “survival probability: single-period difference.” The following is true for each of the three SVRRs and for VSL: (1) its comparative statics (unchanged, increasing, decreasing, or ambiguous) are *not* the same for past, current, and future-period differences in income, and moreover (2) its comparative statics are *not* the same for current and future-period differences in survival probability.

Second, the prioritarian SVRRs, ex ante and ex post, are history-dependent—specifically, with respect to income. Each is decreasing with a one-period change to past income—by contrast with the utilitarian SVRR and VSL, which are independent of past income.

Third, this table confirms a key finding of Adler et al. (2014), using a simpler, single-period model: the manner in which VSL values risk reduction is *not* robust to a change in social evaluation framework. VSL differs, in some significant way, from *each* of the SVRRs. VSL and the utilitarian SVRR have the same comparative statics with respect to income, but not survival probability. VSL and the prioritarian SVRRs have different comparative statics with respect to both income and survival probability.^35^

Fourth, the choice *within* the prioritarian family, between ex ante and ex post prioritarian approaches, is seen to be significant. The ex ante prioritarian SVRR is decreasing in current survival probability and ambiguous with respect to future survival probability, while the ex post prioritarian SVRR is independent of current survival probability and increasing in future survival probability. Both SVRRs are decreasing in past income, but: the ex ante prioritarian SVRR is ambiguous with respect to current and future income, while the ex post prioritarian SVRR is *increasing* with current and future income.^36^

This table, of course, concerns comparative statics: the direction of impact on VSL and the SVRRs of changes in risk and survival probability. It doesn’t show the magnitude of impact—another type of difference between the various approaches. This difference will emerge in the following section, where we empirically estimate VSL and the SVRRs for the U.S. population.

## 5 SVRRs and VSL for the U.S. population

In this Section, we illustrate the SVRR and VSL concepts, and estimate their relative magnitudes, by calculating SVRR and VSL for cohorts of individuals characterized by varying risk profiles, income profiles and ages. The income and survival data for this exercise derive from the actual U.S. population. The U.S. Census Bureau collects data on the income distribution by age range. We used this to estimate the percentiles of the income distribution for each age. Assuming zero mobility (movement across percentiles), we determined a lifetime income profile for the 10^th^, 30^th^, 50^th^, 70^th^, and 90^th^ percentiles of the U.S. income distribution.

The lifetime risk profile for each of these five percentiles was based upon the actual U.S. population survival curve, and then adjusted to reflect income differences in life expectancy.^37^

We calculated the utilitarian SVRR, ex ante and ex post prioritarian SVRRs, and VSL by age for each of the five percentiles. As per the analysis in Sections 2,3 and 4, we did so on the assumption that an individual’s annual consumption in a given year is just her income. A logarithmic utility function was used.^38^ The utility discount rate was set to 0. For the prioritarian SVRRs, we used an “Atkinson” (isoelastic) SWF with both a moderate inequality-aversion parameter (γ = 1) and a higher such parameter (γ = 2). This yields four different prioritarian SVRRs (namely ex post or ex ante, with γ = 1 or 2). (On the attractive axiomatic properties of the Atkinson subfamily of prioritarian SWFs, see Adler, 2012, ch. 5; Adler, 2019, pp. 154–58.)

**Fig. 1:**
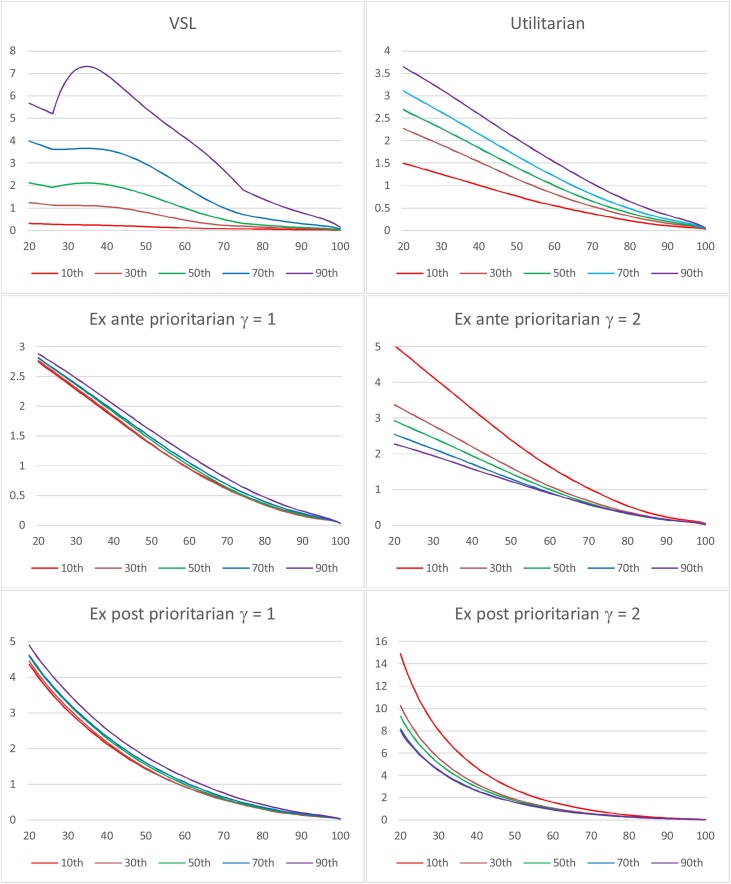
SVRRs and VSL by Age and Income Percentile for the U.S. Population.

The panels in Fig. 1 display the SVRRs and VSL as a function of age, for each of the five percentiles. The results are normalized so that 1 represents the SVRR or VSL for a 60 year old, median income individual.

As the panels show, the utilitarian SVRR decreases with age within each percentile (even though this is not theoretically required—see Section 3.1). The prioritarian SVRRs also decrease with age within each percentile (as is required by Priority for the Young and Ratio Priority for the Young given that the utilitarian SVRR does^39^).

The utilitarian SVRR increases with income: at every age, individuals in higher income percentiles have larger SVRRs. This is reversed for the prioritarian SVRRs with γ = 2; at every age, SVRR decreases with income. γ = 1 is an intermediate case, in which the utilitarian preference for reducing the risk of those with higher income is almost neutralized but not reversed. Note here that the lines displaying the ex ante and ex post prioritarian SVRR as a function of age are virtually the same for all income percentiles. Thus the prioritarian SVRRs with moderate inequality aversion conform to widely held views regarding lifesaving policies, namely that the young should take priority but income should make no difference.^40^

VSL decreases with age for individuals above 40. At earlier ages, for some income percentiles, VSL displays the inverted U (“hump”) shape often described in the literature. (Aldy and Viscusi, 2007; Hammitt, 2007; Viscusi, 2018, ch. 5).

**Fig. 2:**
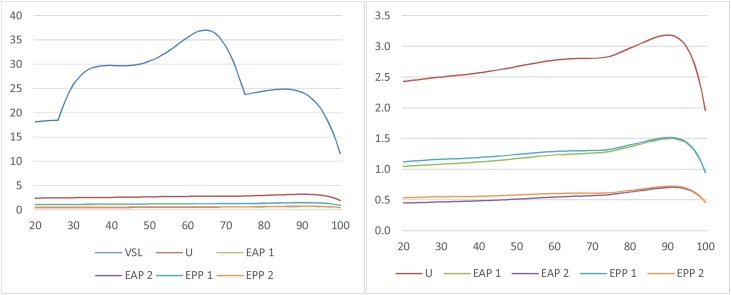
Ratio of SVRRs and VSL at 90^th^ Percentile Income to SVRRs and VSL at 10^th^ Percentile Income by Age.

**Fig. 3:**
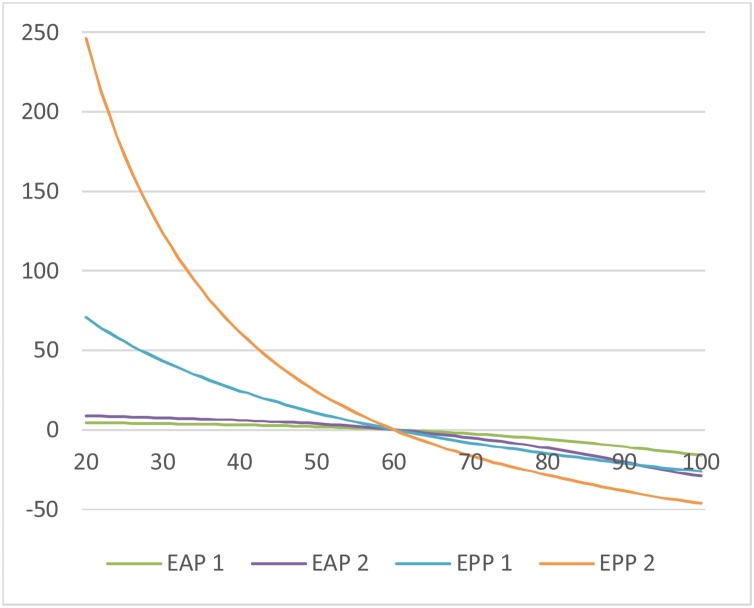
Fair Innings Premium (percent).

The most striking difference between VSL and all the SVRRs concerns income effects: VSL increases with income at all ages, and much more steeply than even the utilitarian SVRR. This can be observed in Fig. 1, and is displayed very clearly in Fig. 2, which shows the ratio between SVRR or VSL at the 90^th^ and 10^th^ income percentiles as a function of age. That ratio is between 0.5 and 3 for all the SVRRs, while generally exceeds 20 for VSL. (In Fig. 2, the abbreviation “U” indicates the utilitarian SVRR; “EAP 1” and “EAP 2” the ex ante prioritarian SVRR with γ = 1 and 2, respectively; and “EPP 1” and “EPP 2” the ex post prioritarian SVRR with γ = 1 and 2, respectively. The “EAP” and “EPP” abbreviations are also used in Fig. 3.)

Our exercise here also sheds light on the U.S. government’s practice of employing a single, population-average VSL to value risk reduction (Robinson, 2007). Such an approach is not only inconsistent with the theory of BCA—as Fig. 1 shows, VSL varies by age and income—but also with the SWF framework. All of the SVRRs vary, at least, by age, and some by both age and income.

Finally (see Fig. 3) we estimate a “fair innings premium.” Recall that both ex ante prioritarian and ex post prioritarian SVRRs have the property of Ratio Priority for the Young: the ratio of prioritarian SVRRs, between a younger and older person with the same lifetime income and risk profile, is always larger than the ratio of utilitarian SVRRs. Fig. 3 shows the magnitude of this difference in ratios. For individuals of the median income profile and associated risk profile, we calculate the percentage by which the ratio between the ex ante or ex post prioritarian SVRR of an individual of each age and a 60-year-old’s ex ante or ex post prioritarian SVRR exceeds the comparable ratio for the utilitarian SVRR.^41^

## 6 Conclusion

This Article has undertaken an extensive analysis of the social value of risk reduction (SVRR). SVRR is the linchpin concept for applying a social welfare function (SWF) to one major policy domain: fatality risk regulation. SVRR*_i_* is defined as $\frac{\partial W}{\partial p_{i}}$. It is the marginal change in social value, as determined by SWF *W*(·), per unit of risk reduction for individual *i*. We investigate SVRR for three major SWFs (utilitarian, ex ante prioritarian, and ex post prioritarian), using a lifetime model that allows us to differentiate individuals by age, lifetime risk profile, and lifetime income profile.

Economists have intensively investigated the SWF framework in certain policy arenas, such as taxation and climate policy. However, the application of SWFs to the domain of risk regulation has been little studied. Our analysis demonstrates, in detail, how the social weight placed upon a reduction in a given individual’s fatality risk depends upon the functional form of the SWF. In their comparative statics with respect to income and baseline risk, the three SVRRs differ significantly from each other. At the same time, each of the SVRRs deviates substantially from VSL—the valuation concept for risk reduction that is used by benefit-cost analysis (BCA), currently the dominant methodology in governmental practice and in applied economics. In a nutshell, then, our analysis shows that a rigorous intellectual apparatus with deep roots in welfare economics—the SWF framework—values individual risk reduction in a manner quite different from BCA. In an empirical exercise, we confirm this finding.

Moreover, we show that the “fair innings” concept, popular in the public health literature, has a firm, formal basis *within* welfare economics. Specifically, both the ex ante prioritarian and ex post prioritarian SVRRs satisfy axioms of Priority for the Young and Ratio Priority for the Young. In effect, a younger person takes priority over an older person with respect to risk reduction even when the gains in expected lifetime well-being are equal. (By contrast, BCA does not support the fair innings concept; a younger individual may have a *lower* VSL even when the gains to expected lifetime well-being are equal.) As far as we are aware, this Article is the first to provide a theoretical interpretation of “fair innings” using the tools of welfare economics.

## Declaration of Competing Interest

The authors report no competing interest.

## Footnotes

5 More precisely, this proposition is hard to dispute for those who endorse *welfarism*: who believe that governmental policies should be evaluated in light of the sum total and distribution of individual well-being. By contrast, non-welfarists might argue that everyone has an equal right to life, and that governments should not differentially value lifesaving on the basis of any individual characteristics (including age). This Article presupposes welfarism. Welfarism is the dominant ethical view in economics, and both of the assessment frameworks we consider in this article—the social-welfare-function framework and benefit-cost-analysis—are versions of welfarism. (On welfarism, see generally Adler (2012, 2019).)

6 See below, Section 3, explaining why BCA does not support fair innings.

7 The utilitarian SVRR also does not display Priority for the Young and Ratio Priority for the Young, but this is true by definition—since these properties are defined relative to a utilitarian baseline. See Section 3.

8 The properties of the ex ante prioritarian and ex post prioritarian SVRRs depend, to some extent, on which concave transformation function is used—embodying the degree of priority for the worse off. Thus, in Section 5, the prioritarian SVRRs with a moderately concave transformation function are flat with income, while the prioritarian SVRRs with a more concave transformation function decrease with income.

9 Note that our definition of individual *i*’s “age” as *A_i_* is slightly different from the colloquial use of the term “age.” If Betty is at the beginning of period 5 of her life, then (colloquially) we would say that her age is 4, not 5. However, we need a natural-language term to refer to *A_i_*, and “age” is the most natural choice. The issue here is purely semantic. Referring to *A_i_* as individual *i*’s “age” rather than “age plus one” makes no difference to our analytic results.

10 See below, note 22, for an explanation why we assume that *A_i_* ≥ 2.

11 A different way to model an individual’s fatality risks over the life course would be to conceptualize *p_i_*(*t*) as the currently known conditional probability of *i*’s surviving period *t*, given that she is alive at the beginning of that period. On this approach, *p_i_*(*t*) = 1 for *t* < *A_i_*, since *i* knows she has survived to the beginning of period *A_i_*. Modelling fatality risks this way would not change our results, since the formulas for the utilitarian, ex ante prioritarian, and ex post prioritarian SVRRs, and for VSL, do not depend upon past survival probabilities; and because currently known survival probabilities for the present and future periods are the same as at-birth survival probabilities (see Appendix). We assume 0 < *p_i_*(*t*) < 1 for all *t* such that 1 < *t* ≤ *T*; and that *p_i_*(1) = 1. As discussed below, note 22, we assume that *A_i_* ≥ 2 for all *i*—that is, that every individual has survived to the end of the first period of her life—and to ensure this we assume *p_i_*(1) = 1. Finally, *p_i_*(*T* + 1) = 0. (This is the probability that *i* survives one more period, given that she has survived to the end of period *T*, the maximum number of possible periods.)

12 Of course, if *t* and *t** are distinct periods, then it may well be the case that *y_i_*(*t*) ≠ *y_i_*(*t**). But the *profile* of incomes (*y_i_*(1), …, *y_i_*(*T*))—specifying, for each period, what the individual will earn at the end of that period if she survives to that point—does not itself change as the individual ages.

13 In the working paper upon which the current Article is based, we do address the topic of SVRR*_i_* with saving and borrowing, although do not undertake a comprehensive analysis. See Adler et al. (2019).

14 Since consumption occurs at the end of each period, the discount rate β is raised to the power *t* rather than (*t*−1).

15 “iff” means “if and only if.”

16 In order to ensure that *u*(*c_i_*(*t*)) > 0 for all *i* and *t*, we assume that there is a “subsistence” level of consumption *c^zero^* such that *u*(*c^zero^*) = 0 and that *y_i_*(*t*) > *c^zero^* for all *i*, *t*.

17 As noted above, if *t* < *A_i_*, then π*_i_*(*t*; *A_i_*) = 1.

18 SVRR*_i_* for a given *W*(·) is the partial derivative of *W*(·) with respect to *i*’s current survival probability. See Section 2.3. In order for this partial derivative to be well-defined, the SWF needs to be score-based, as are *W^U^*, *W^EAP^*, and *W^EPP^* (assigning a real number to each policy and ranking policies in the order of those numbers), and indeed differentiable, as are *W^U^*, *W^EAP^*, and *W^EPP^* given the model here.

19 The choice between ex ante and ex post approaches to equity has also been discussed in health economics. (Bleichrodt, 1997).

20 The rule for ex post prioritarianism is often stated in a different way: as the expected value of the sum of individuals’ transformed well-being. But this is mathematically equivalent to the rule stated in the text: the expected value of the sum of individuals’ transformed well-being equals the sum of individuals’ expected transformed well-being. (Adler, 2019, app. J).

21 The ex ante Pareto axioms are Ex Ante Pareto Indifference and Ex Ante Strong Pareto. Ex Ante Pareto Indifference: If each person’s expected well-being with policy *P* is equal to her expected well-being with policy *P**, then *P* and *P** are equally good. Ex Ante Strong Pareto: If each person’s expected well-being with policy *P* is greater than or equal to her expected well-being with policy *P**, and at least one person’s expected well-being is strictly greater, then *P* is better than *P**. Stochastic Dominance: If, for each possible state of nature, the well-being vector produced by policy *P* in that state is better than the well-being vector produced by policy *P**, then *P* is better than *P**. On the axiomatic properties of utilitarianism and prioritarianism under uncertainty, see generally Adler, 2012 ch. 7; Adler, 2019 ch. 3–4, apps. I-L.

22 The “Atkinson” family of *g*(·) functions—*g*(*u*) = (1−γ)^−1^*u*^1−γ^, γ > 0, γ ≠ 1; and *g*(*u*) = ln *u* if γ = 1—have attractive axiomatic properties and are regularly used in the economic literature on prioritarianism. See Adler (2012, ch. 5). (Indeed our empirical exercise in Section 5 uses an Atkinson *g*(·) function.) The Atkinson *g*(·) is such that *g*(0) is undefined for γ ≥ 1. In order for our analysis to accommodate the possibility that *g*(0) is undefined, we assume that *A_i_* ≥ 2 for all *i*. (Note that the expression for the ex post prioritarian SWF in Definition 1c includes the *g*(·) value of individual *i*’s lifetime well-being if she lives exactly (*A_i_* − 1) periods. If *A_i_* = 1, the individual’s lifetime well-being if she lives exactly *A_i_* − 1 periods is 0.) Because the period length can be arbitrarily short, the assumption that *A_i_* ≥ 2 is not significantly restrictive.

23 Similarly, while much empirical work on income inequality focuses on annual income, there is also a significant body of work that looks at the inequality of lifetime income. See, for example, Bönke et al. (2015); Bowlus and Robin (2004); Guvenen et al. (2017); Huggett et al. (2011); Nilsen et al. (2012).

24 Formally,$SVRR_{i}=\frac{\partial W}{\partial p_{i}(A_{i})}(p_{i}^{O}(1),...,p_{i}^{O}(T);y_{i}^{O}(1),...,y_{i}^{O}(T))$. Since all three SWFs are additively separable across individuals, $\frac{\partial W}{\partial p_{i}(A_{i})}$ can be expressed just as a function of *i*’s risk and income profiles; the value of this partial derivative does not depend upon other individuals’ risks and incomes.

25 Assume that a policy intervention changes individual *i*’s current survival probability by Δ*p_i_*; her survival probability in period *t* by Δ*p_i_^t^*, with *t* > *A_i_*; and her income in period *t* by Δ*y_i_^t^*, with *t* ≥ *A_i_*. Then, by the total-differential approximation from calculus, Δ*W* is approximately equal to: $\sum_{i}\left(SVRR_{i}\times \Delta p_{i}+\sum_{t=A_{i}+1}^{T}\frac{\partial W}{\partial p_{i}(t)}(p_{i}^{O}(1),...,p_{i}^{O}(T);y_{i}^{O}(1),...,y_{i}^{O}(T))\times \Delta p_{i}^{t}+\sum_{t=A_{i}}^{T}\frac{\partial W}{\partial y_{i}(t)}(p_{i}^{O}(1),...,p_{i}^{O}(T);y_{i}^{O}(1),...,y_{i}^{O}(T))\times \Delta y_{i}^{t}\right)$

26 We remind the reader that proofs of numbered propositions can be found in the on-line Appendix.

27 Let $V_{i}(p_{i}(1),...,p_{i}(T);y_{i}(1),...,y_{i}(T))$ denote individual *i’s* expected lifetime utility as a function of risk profile (*p_i_*(1), …, *p_i_*(*T*)) and income profile (*y_i_*(1), …, *y_i_*(*T*)). That is, $V_{i}(p_{i}(1),...,p_{i}(T);y_{i}(1),...,y_{i}(T))=\sum_{t=1}^{A_{i}-1}\beta^{t}u(y_{i}(t))+\sum_{t=A_{i}}^{T}\pi_{i}(t;A_{i})\beta^{t}u(y_{i}(t))$, with $\pi_{i}(t;A_{i})=\prod_{s=A_{i}}^{t}p_{i}(s)$. Then ME*_i_*(*P*) = Δ*y* such that: $V_{i}(p_{i}^{O}(1),...,p_{i}^{O}(T);y_{i}^{O}(1),...,y_{i}^{O}(A_{i}-1),y_{i}^{O}(A_{i})+\Delta y,y_{i}^{O}(A_{i}+1),...,y_{i}^{O}(T))=V_{i}(p_{i}^{P}(1),...,p_{i}^{P}(T);y_{i}^{P}(1),...,y_{i}^{P}(A_{i}),...,y_{i}^{p}(T))$ Note that *ME_i_*(*P*), thus defined, is individual *i*’s “equivalent variation” for policy *P*. Sometimes, BCA is defined instead as the sum of “compensating variations.” (Boadway, 2016; Freeman, 2003, ch. 3.) There are theoretical advantages to conceptualizing monetary equivalents for purposes of BCA as equivalent variations rather than compensating variations. (Adler et al., 2014, n. 8.) However, that choice is not significant for purpose of this Article. Whether monetary equivalents are defined as equivalent variations or compensating variations, $VSL_{i}=\frac{\partial B}{\partial p_{i}(A_{i})}$. See Appendix.

28 To state this definition more formally, let *V_i_* be expressed as a function of individual *i*’s risk profile and income profile, as in note 27 immediately above. $V_{i}=V_{i}({p_{i}}^{}(1),...,p_{i}(T);{y_{i}}^{}(1),...,y_{i}(T))$. Then VSL*_i_* = $\frac{\partial V_{i}}{\partial p_{i}(A_{i})}(p_{i}^{O}(1),...,p_{i}^{O}(T);y_{i}^{O}(1),...,y_{i}^{O}(T))/\frac{\partial V_{i}}{\partial y_{i}(A_{i})}(p_{i}^{O}(1),...,p_{i}^{O}(T);y_{i}^{O}(1),...,y_{i}^{O}(T))$. Adler et al. (2014), using a one-period model, defined VSL as the marginal rate of substitution between wealth and survival probability. In the multi-period model with myopic consumption that we use in this Article, an individual’s wealth in each period is zero—she consumes what she earns and saves nothing—and so VSL is here defined as the marginal rate of substitution between income (or equivalently, consumption) and survival probability.

29 As in note 25 above, consider a policy intervention that changes individual *i*’s current survival probability by Δ*p_i_*; her survival probability in period *t* by Δ*p_i_^t^*, with *t* > *A_i_*; and her income in period *t* by Δ*y_i_^t^*, with *t* ≥ *A_i_*. Again using the total-differential approximation from calculus, Δ*B* is approximately equal to: $\sum_{i}\left(VSL_{i}\times \Delta p_{i}+\sum_{t=A_{i}+1}^{T}\frac{\partial B}{\partial p_{i}(t)}(p_{i}^{O}(1),...,p_{i}^{O}(T);y_{i}^{O}(1),...,y_{i}^{O}(T))\times \Delta p_{i}^{t}+\sum_{t=A_{i}}^{T}\frac{\partial B}{\partial y_{i}(t)}(p_{i}^{O}(1),...,p_{i}^{O}(T);y_{i}^{O}(1),...,y_{i}^{O}(T))\times \Delta y_{i}^{t}\right)$. The terms in this equation for the partial derivative of *B*(·) with respect to survival probability in a future period *t*—that is $\frac{\partial B}{\partial p_{i}(t)}$, *t* > *A_i_*—can also be related to VSL*_i_*. It can be shown that each such term equals VSL*_i_* multiplied by the marginal rate of substitution with respect to *V_i_* between present survival probability and survival probability in period *t*.

30 *V_j_*, the expected lifetime well-being of the younger individual, is (1 − *p*_2_) (1) + *p*_2_(1 − *p*_3_) (2) + *p_2_p_3_*(3) = 1 + *p*_2_ + *p*_2_*p*_3_; while *V_i_*, the expected lifetime well-being of the older individual, is (1 − *p*_3_) (2) + *p*_3_(3) = 2 + *p*_3_. Thus, $S_{j}^{U}=\frac{\partial V_{j}}{\partial p_{2}}=1+p_{3}$, while $S_{i}^{U}=\frac{\partial V_{i}}{\partial p_{3}}=1$. $S_{j}^{EAP}=g^{\prime }(1+p_{2}+p_{2}p_{3})(1+p_{3})$and $S_{i}^{EAP}=g^{\prime }(2+p_{3})$.

31 In what follows, we assume that SVRR values are always positive. (As remarked earlier, this is true, given the assumptions of our model, for $S_{i}^{U}$, $S_{i}^{EAP}$, and $S_{i}^{EPP}$; see Section 2.3.) First, Ratio Priority for the Young implies Priority for the Young. (1) If $S_{j}^{U}-S_{i}^{U}>0$, then $S_{j}^{U}/S_{i}^{U}>1$. By Ratio Priority for the Young, *S_j_*/*S_i_*>1. Thus *S_j_ *− *S_i_*>0. (2) If $S_{j}^{U}-S_{i}^{U}=0$, then $S_{j}^{U}/S_{i}^{U}=1$. By Ratio Priority for the Young, *S_j_*/*S_i_*>1. Thus *S_j_ *− *S_i_*>0. Second, Priority for the Young does not imply Ratio Priority for the Young. To see this, consider the case in which $S_{j}^{U}-S_{i}^{U}<0$. In this case, Priority for the Young places no constraint on the relative magnitudes of *S_j_* and *S_i_*. However, Ratio Priority for the Young requires that ${S_{j}}^{}/{S_{i}}^{}>S_{j}^{U}/S_{i}^{U}$. It’s also true, by directly parallel reasoning, that Ratio Priority for the Young as defined with respect to VSL is logically stronger than Priority for the Young as defined with respect to VSL. If VSL were to satisfy the first, it would satisfy the second, but not vice versa. As it happens, however, VSL does not satisfy either. See Section 3.4.

32 Let *G_j_* and *G_i_* denote each individual’s expected transformed lifetime well-being. *G_j_* = (1−*p*_2_)*g*(1) + *p*_2_(1−*p*_3_)*g*(2) + *p*_2_*p*_3_*g*(3). *G_i_* = (1−*p*_3_)*g*(2) + *p*_3_*g*(3). Then $S_{j}^{EPP}=\frac{\partial G_{j}}{\partial p_{2}}=[g(2)-g(1)]+p_{3}[g(3)-g(2)]$, while $S_{i}^{EPP}=\frac{\partial G_{i}}{\partial p_{3}}=g(3)-g(2)$.

33 See note 31.

34 By “ambiguous” we mean the following. The comparative statics of SVRR or VSL with respect to a parameter of interest (present income, future income, permanent income, age, etc.) are “ambiguous” if (a) we can find some combination of the other parameters and strictly increasing and strictly concave *u*(·) and *g*(·) such that SVRR or VSL is increasing in the parameter of interest, and (b) some alternative combination of the other parameters and the same *u*(·) and *g*(·) such that SVRR or VSL is decreasing in the parameter of interest.

35 Except that, if g(·) has a coefficient of relative risk aversion less than or equal to one, the comparative statics of the ex ante prioritarian SVRR with respect to survival probability are the same as VSL.

36 See Adler and Treich (2017), finding significant differences between ex ante and ex post prioritarianism in a model of intergenerational consumption allocation.

37 Specifically, data on the U.S. income distribution was taken from the Current Population Survey (CPS) income tables. See https://www.census.gov/data/tables/time-series/demo/income-poverty/cps-pinc.html. We used the table PINC_01_1_1_1 (total work experience, both sexes, all races) for 2016. A fourth degree polynomial in age was fitted to the data. We assume that income for ages 20−24 is the same as for ages 25−30 and that income for ages 75−100 is the same as for ages 70−74. The U.S. population survival curve was taken from the life tables compiled by the Centers for Disease Control and Prevention. See https://www.cdc.gov/nchs/products/life_tables.htm. We used the 2014 life tables (National Vital Statistics Reports, vol. 66, no. 4, August 24, 2017). A lifetime risk profile for the five percentiles was determined as follows. We adjusted the U.S. population survival curve, as taken from the life table, by multiplying the annual mortality risk for each age by a scaling factor to reflect the individual’s income. These scaling factors were, respectively, 1.5, 1.2, 1, 0.9, and 0.7 for, respectively, the 10^th^, 30^th^, 50^th^, 70^th^, and 90^th^ percentiles of income. The scaling factors were taken from Adler (2017, appendix C), who in turn estimated them to match findings by Chetty et al. (2016) regarding differences in life expectancy across income classes.

38 Specifically, *u*(*c*) = ln *c* − ln *c^zero^*, *c^zero^* = $1000, which is roughly the World Bank level of extreme poverty. See Adler (2017, appendix C).

39 Assume that the utilitarian SVRR decreases with age among a group of individuals with the same risk and income profile. That is, for every age *A*, if *A_j_* = *A* and *A_i_* = *A* + 1 and *i* and *j* are in the group, then $S_{j}^{U}>S_{i}^{U}$. Since the prioritarian SVRRs satisfy Priority for the Young, it follows that $S_{j}^{EAP}>S_{i}^{EAP}$ and $S_{j}^{EPP}>S_{i}^{EPP}$. Because Ratio Priority for the Young implies Priority for the Young (see note 31), this also follows from Ratio Priority for the Young.

40 Dolan et al. (2005, p. 202), reviewing the survey literature regarding the public’s preferences for the allocation of health care, finds that “most studies suggest that health gains to the old are weighted less.” Dolan et al. find some studies in which respondents prefer to allocate resources to those in lower socioeconomic groups. However, Emanuel et al. (2020, p. 2051), discussing the allocation of scarce medical resources in the Covid-19 pandemic, and citing the public health literature, write: “Consensus exists that an individual person’s wealth should not determine who lives or dies.”

41 That is, we calculate $[(S_{j}^{EAP}/S_{60}^{EAP})/(S_{j}^{U}/S_{60}^{U})]-1$ and $[(S_{j}^{EPP}/S_{60}^{EPP})/(S_{j}^{U}/S_{60}^{U})]-1$ for each age *j*.
