# Supplementary material for: Fair innings? The utilitarian and prioritarian value of risk reduction over a whole lifetime
Source: J Health Econ. 2021 Jan;75:102412. doi: 10.1016/j.jhealeco.2020.102412 (PMC7846816; doi:10.1016/j.jhealeco.2020.102412)
Supplement: Supplementary file 1 [file mmc1.pdf]

**On-line Appendix for Adler, Ferranna, Hammitt, Treich, "Fair Innings? The Utilitarian and Prioritarian Value of Risk Reduction over a Whole Lifetime."**

This Appendix provides mathematical backup for all propositions stated in the text of the Article, and for other mathematical claims whose derivations are not straightforward. The order of the Appendix corresponds to the order of the text.

Formulas for $\pi_i(t; t^*)$ and $\mu_i(t; t^*)$ are used pervasively in the derivations. As stated in the text, $\pi_i(t; t^*)$ is individual $i$'s probability of surviving through the end of period $t$, conditional on being alive at the beginning of period $t^*$. In particular, $\pi_i(t; A_i)$ is individual $i$'s probability of surviving through the end of period $t$, conditional on her current age ($A_i$). If $t < t^*$, then $\pi_i(t; t^*) = 1$. If $t \geq t^*$, then $\pi_i(t;t^*) = \prod_{s=t^*}^{t} p_i(s)$. Substituting $A_i$ for $t^*$, we arrive at formulas for $\pi_i(t; A_i)$.

It is possible that $A_i = T$. In this case, it is not clear how to interpret $\pi_i(t; A_i + 1)$, $t \leq T$. We stipulate that $\pi_i(t; A_i + 1) = 1$ if $A_i = T$ and $t \leq T$.[1]

$\mu_i(t; t^*)$ is individual $i$'s probability of surviving through the end of period $t$ and then dying—the probability of living exactly $t$ periods—conditional on being alive at the beginning of period $t^*$. In particular, $\mu_i(t; A_i)$ is individual $i$'s probability of living exactly $t$ periods, conditional on her current age. If $t < t^* - 1$, $\mu_i(t; t^*) = 0$. If $t = t^* - 1$, $\mu_i(t; t^*) = 1 - p_i(t^*)$. If $t \geq t^*$, $\mu_i(t;t^*) = \left( \prod_{s=t^*}^{t} p_i(s) \right)(1 - p_i(t+1)) = \pi_i(t;t^*)(1 - p_i(t+1))$. Substituting $A_i$ for $t^*$, we arrive at formulas for $\mu_i(t; A_i)$.

It is possible that $A_i = T$. In this case, we stipulate that $\mu_i(t; A_i + 1) = 1$ if $t = T$ and $\mu_i(t; A_i + 1) = 0$ otherwise.

The derivations often use the summation symbol, $\sum$. If the variables are such that the lower bound of the summation exceeds the upper bound, then the summation is "empty" and set equal to 0. For example, if $A_i = T$, then the summation $\sum_{t=A_i+1}^{T} f(t)$ equals zero. The derivations also use the product symbol $\prod$. If the lower bound of the product exceeds the upper bound, then the product is "empty" and set equal to 1. For example, if $t = A_i$, the product $\prod_{s=A_i+1}^{t} f(s)$ equals 1.

[1] Individuals live at most $T$ periods, and so the supposition that individual $i$ is alive at the beginning of period $T + 1$ may be thought to be problematic. Actually, if we conceptualize the beginning of period $T + 1$ as the moment in time right after the end of period $T$, then $\pi_i(t; T + 1)$, $t \leq T$, is individual $i$'s probability of surviving through the end of period $t$, conditional on having survived to the end of period $T$, which is 1. In any event, to avoid doubt on the issue, we simply stipulate that $\pi_i(t; A_i + 1) = 1$ if $A_i = T$ and $t \leq T$.

## I. Results in Section 2 of the Article

Section 2.1: Relation between At-Birth Survival Probabilities and Currently Known Survival Probabilities.

As explained in Section 2.1, individual $i$'s profile of survival probabilities ($p_i(1), \ldots, p_i(T)$) are her at-birth probabilities, which do not change as she ages. It bears a reminder that these at-birth "survival probabilities" are conditional probabilities: $p_i(t)$ is individual $i$'s probability of surviving to the end of period $t$, conditional on being alive at the beginning of period $t$. The key mathematical concepts that drive the Article's analysis—namely, the utilitarian, ex ante prioritarian, and ex post prioritarian SWFs (Definitions 1a, 1b, 1c); the $\text{SVRR}_i$ defined in terms of these SWFs (Definition 2); and $\text{VSL}_i$ (Definition 4)—are all defined in terms of individuals' profiles of at-birth survival probabilities.

In note 11, we comment that our analysis would not change if we used currently-known survival probabilities rather than at-birth survival probabilities. Let ($p_i^*(1), \ldots, p_i^*(T)$) be individual $i$'s profile of survival probabilities, updated to reflect the information that she has survived to the beginning of the current period, period $A_i$ in her life. Note that $p_i^*(t) = \pi_i(t; A_i)/\pi_i(t-1; A_i)$. If $t < A_i$, then $\pi_i(t; A_i)/\pi_i(t-1; A_i) = 1/1 = 1$. If $t = A_i$, then $\pi_i(t;A_i)/\pi_i(t-1;A_i) = p_i(A_i)/1 = p_i(A_i)$. If $t > A_i$, then

$$\pi_i(t;A_i)/\pi_i(t-1;A_i) = \left(\prod_{s=A_i}^{t} p_i(s)\right) / \left(\prod_{s=A_i}^{t-1} p_i(s)\right) = p_i(t).$$

Thus $p_i^*(t) = 1$ for $t < A_i$, and $p_i^*(t) = p_i(t)$ for $t \geq A_i$. Finally, it can be observed that the SWFs, $\text{SVRR}_i$, and $\text{VSL}_i$ are all defined in terms of individuals' survival probabilities for the present and future periods, not past periods. The values of $W^U$, $W^{EAP}$, $W^{EPP}$, the corresponding SVRRs, and $\text{VSL}_i$ do not depend upon $p_i(t)$ for $t < A_i$. Because $p_i^*(t) = p_i(t)$ for $t \geq A_i$, our analysis would reach the same result if we defined our key concepts (SWFs, $\text{SVRR}_i$, and $\text{VSL}_i$) in terms of currently known survival probabilities rather than at-birth survival probabilities.

Section 2.1: Alternative Formula for $V_i$. $V_i = \sum_{t=1}^{A_i-1} \beta^t u(y_i(t)) + \sum_{t=A_i}^{T} \pi_i(t;A_i)\beta^t u(y_i(t))$

$V_i$ is $i$'s expected lifetime well-being with a given income and risk profile. Thus the main formula for $V_i$ is $\sum_{t=A_i-1}^{T} \mu_i(t;A_i)U_i(t)$.

$$\sum_{t=A_i-1}^{T} \mu_i(t;A_i)U_i(t) = (1-p_i(A_i))\sum_{s=1}^{A_i-1}\beta^s u(y_i(s)) + \sum_{t=A_i}^{T}\pi_i(t;A_i)(1-p_i(t+1))\sum_{s=1}^{t}\beta^s u(y_i(s))$$

$= \sum_{s=1}^{A_i-1}\beta^s u(y_i(s)) + \sum_{t=A_i}^{T}\pi_i(t;A_i)\left(\sum_{s=1}^{t}\beta^s u(y_i(s)) - \sum_{s=1}^{t-1}\beta^s u(y_i(s))\right)$. To see this, note that $\pi_i(t; A_i)p_i(t+1) = \pi_i(t+1; A_i)$ and that $p_i(T+1) = 0$. In turn,

$$\sum_{t=A_i}^{T}\pi_i(t;A_i)\left(\sum_{s=1}^{t}\beta^s u(y_i(s))-\sum_{s=1}^{t-1}\beta^s u(y_i(s))\right)=\sum_{t=A_i}^{T}\pi_i(t;A_i)\beta^t u(y_i(t)).$$

Proposition 1a. $S_i^U = -U_i^O(A_i-1)+\sum_{t=A_i}^{T}\frac{\mu_i^O(t;A_i)}{p_i^O(A_i)}U_i^O(t)$

This proposition follows from the definition of $V_i$ as $\sum_{t=A_i-1}^{T}\mu_i(t;A_i)U_i(t)$; the definition of $W^U(\cdot)$ as the sum of $V_i$ (Definition 1a); and the definition of SVRR$_i$ as the partial derivative of $W(\cdot)$ with respect to $i$'s current survival probability $p_i(A_i)$, evaluated at $i$'s status quo income and risk profile (Definition 2). Note that $\mu_i(A_i-1;A_i)=1-p_i(A_i)$, and that $\mu_i(t;A_i)=\left(\prod_{s=A_i}^{t}p_i(s)\right)(1-p_i(t+1))$ for $t\geq A_i$. Thus $\frac{\partial\mu_i(t;A_i)}{\partial p_i(A_i)}=-1$ for $t=A_i-1$; and $\frac{\partial\mu_i(t;A_i)}{\partial p_i(A_i)}=\frac{\mu_i(t;A_i)}{p_i(A_i)}$ for $t\geq A_i$.

Proposition 1b. $S_i^{EAP}=g'(V_i^O)S_i^U$

This proposition follows from the definition of $W^{EAP}(\cdot)$ as the sum of $g(V_i)$ (Definition 1b), the definition of SVRR$_i$, and the observation that $S_i^U$ is equal $\frac{\partial V_i}{\partial p_i(A_i)}$ evaluated at $i$'s status quo risk and income profile.

Proposition 1c. $S_i^{EPP}=-g(U_i^O(A_i-1))+\sum_{t=A_i}^{T}\frac{\mu_i^O(t;A_i)}{p_i^O(A_i)}g(U_i^O(t))$

This proposition follows from the definition of $W^{EPP}$ as the sum across individuals of $\sum_{t=A_i-1}^{T}\mu_i(t;A_i)g(U_i(t))$ (Definition 1c) and the definition of SVRR$_i$. The derivation is analogous to the derivation of Proposition 1a.

Proposition 2a. $VSL_i=\frac{\partial B}{\partial p_i(A_i)}$, with $\frac{\partial B}{\partial p_i(A_i)}$ evauated at $i$'s status quo risk and income profiles.

Let $(\mathbf{p}_i^P, \mathbf{y}_i^P)$ be shorthand for $i$'s risk and income profile with policy $P$. $ME_i(P)$ = $ME_i(\mathbf{p}_i^P, \mathbf{y}_i^P)$ is such that:

$$V_i(p_i^O(1),..., p_i^O(T); y_i^O(1),..., y_i^O(A_i-1), y_i^O(A_i)+ME_i(\mathbf{p}_i^P, \mathbf{y}_i^P), y_i^O(A_i+1),..., y_i^O(T)) = V_i(\mathbf{p}_i^P, \mathbf{y}_i^P).$$

In what follows, we assume that $ME_i(\cdot)$ is a differentiable function of $i$'s risk and income profile.

Let $F(ME_i(\mathbf{p}_i^P, \mathbf{y}_i^P)) =$

$$V_i(p_i^O(1),..., p_i^O(T); y_i^O(1),..., y_i^O(A_i-1), y_i^O(A_i)+ME_i(\mathbf{p}_i^P, \mathbf{y}_i^P), y_i^O(A_i+1),..., y_i^O(T))$$

Then $F(ME_i(\mathbf{p}_i^P, \mathbf{y}_i^P)) = V_i(\mathbf{p}_i^P, \mathbf{y}_i^P)$. Differentiating both sides by $p_i(A_i)$, we have that $\frac{dF}{dME_i}(ME_i(\mathbf{p}_i^P, \mathbf{y}_i^P))\frac{\partial ME_i}{\partial p_i(A_i)}(\mathbf{p}_i^P, \mathbf{y}_i^P) = \frac{\partial V_i}{\partial p_i(A_i)}(\mathbf{p}_i^P, \mathbf{y}_i^P)$ . In particular, $\frac{dF}{dME_i}(ME_i(\mathbf{p}_i^O, \mathbf{y}_i^O))\frac{\partial ME_i}{\partial p_i(A_i)}(\mathbf{p}_i^O, \mathbf{y}_i^O) = \frac{\partial V_i}{\partial p_i(A_i)}(\mathbf{p}_i^O, \mathbf{y}_i^O)$. Note now that $ME_i(\mathbf{p}_i^O, \mathbf{y}_i^O) = 0$ and thus $\frac{dF}{dME_i}(ME_i(\mathbf{p}_i^O, \mathbf{y}_i^O)) = \frac{\partial V_i}{\partial y_i(A_i)}(\mathbf{p}_i^O, \mathbf{y}_i^O)$. Thus $\frac{\partial ME_i}{\partial p_i(A_i)}(\mathbf{p}_i^O, \mathbf{y}_i^O) = \frac{\partial V_i}{\partial p_i(A_i)}(\mathbf{p}_i^O, \mathbf{y}_i^O) / \frac{\partial V_i}{\partial y_i(A_i)}(\mathbf{p}_i^O, \mathbf{y}_i^O) = VSL_i$ . Finally, since $B((\mathbf{p}_1^P, \mathbf{y}_1^P),...,(\mathbf{p}_N^P, \mathbf{y}_N^P)) = \sum_{i=1}^N ME_i((\mathbf{p}_i^P, \mathbf{y}_i^P)$ , we have that $\frac{\partial B}{\partial p_i(A_i)}((\mathbf{p}_1^O, \mathbf{y}_1^O),...,(\mathbf{p}_N^O, \mathbf{y}_N^O)) = \frac{\partial ME_i}{\partial p_i(A_i)}(\mathbf{p}_i^O, \mathbf{y}_i^O) = VSL_i$.

In note 27 of the Article, we note that it is also the case that $VSL_i = \frac{\partial B}{\partial p_i(A_i)}$ if $B$ is defined in terms of compensating rather than equivalent variations. Let individual $i$'s compensating variation, $CV_i(\mathbf{p}_i^P, \mathbf{y}_i^P)$, be defined as follows. $CV_i(\mathbf{p}_i^P, \mathbf{y}_i^P)$ is such that: $V_i(\mathbf{p}_i^O, \mathbf{y}_i^O) = V_i(p_i^P(1),..., p_i^P(T); y_i^P(1),..., y_i^P(A_i-1), y_i^P(A_i)-CV_i(\mathbf{p}_i^P, \mathbf{y}_i^P), y_i^P(A_i+1),..., y_i^P(T))$. If $B((\mathbf{p}_1^P, \mathbf{y}_1^P),...,(\mathbf{p}_N^P, \mathbf{y}_N^P)) = \sum_{i=1}^N CV_i((\mathbf{p}_i^P, \mathbf{y}_i^P)$, then $\frac{\partial B}{\partial p_i(A_i)}((\mathbf{p}_1^P, \mathbf{y}_1^P),...,(\mathbf{p}_N^P, \mathbf{y}_N^P)) = \frac{\partial CV_i}{\partial p_i(A_i)}(\mathbf{p}_i^P, \mathbf{y}_i^P)$. We assume, analogously to above, that $CV_i(\cdot)$ is a differentiable function of $i$'s risk and income profile.

Let

$$G(\mathbf{p}_i^P, \mathbf{y}_i^P, CV_i(\mathbf{p}_i^P, \mathbf{y}_i^P)) = V_i(p_i^P(1),..., p_i^P(T); y_i^P(1),..., y_i^P(A_i-1), y_i^P(A_i)-CV_i(\mathbf{p}_i^P, \mathbf{y}_i^P), y_i^P(A_i+1),..., y_i^P(T))$$

. Then $G(\mathbf{p}_i^P, \mathbf{y}_i^P, CV_i(\mathbf{p}_i^P, \mathbf{y}_i^P)) = V_i(\mathbf{p}_i^O, \mathbf{y}_i^O)$. Differenting both sides of this equation with respect to $p_i(A_i)$, we have that $\frac{\partial G}{\partial p_i(A_i)}(\mathbf{p}_i^P, \mathbf{y}_i^P, CV_i(\mathbf{p}_i^P, \mathbf{y}_i^P)) = 0$.

$\frac{\partial G}{\partial p_i(A_i)}(\mathbf{p}_i^P,\mathbf{y}_i^P,CV_i(\mathbf{p}_i^P,\mathbf{y}_i^P))=\frac{\partial V_i}{\partial p_i(A_i)}+\frac{\partial V_i}{\partial CV_i}\frac{\partial CV_i}{\partial p_i(A_i)}$ , with the partial derivatives on the RHS of this equation evaluated at $V_i(p_i^P(1),...,p_i^P(T);y_i^P(1),...,y_i^P(A_i-1),y_i^P(A_i)-CV_i(\mathbf{p}_i^P,\mathbf{y}_i^P),y_i^P(A_i+1),...,y_i^P(T)))$. In particular, noting that $CV_i(\mathbf{p}_i^O,\mathbf{y}_i^O)=0$, we have that $\frac{\partial V_i}{\partial p_i(A_i)}(\mathbf{p}_i^O,\mathbf{y}_i^O)+\frac{\partial V_i}{\partial CV_i}(\mathbf{p}_i^O,\mathbf{y}_i^O)\frac{\partial CV_i}{\partial p_i(A_i)}(\mathbf{p}_i^O,\mathbf{y}_i^O)=0$. Because $\frac{\partial V_i}{\partial CV_i}(\mathbf{p}_i^O,\mathbf{y}_i^O)=-\frac{\partial V_i}{\partial y_i(A_i)}(\mathbf{p}_i^O,\mathbf{y}_i^O)$ , we have that

$\frac{\partial CV_i}{\partial p_i(A_i)}(\mathbf{p}_i^O,\mathbf{y}_i^O)=\frac{\partial V_i}{\partial p_i(A_i)}(\mathbf{p}_i^O,\mathbf{y}_i^O)/\frac{\partial V_i}{\partial y_i(A_i)}(\mathbf{p}_i^O,\mathbf{y}_i^O)=VSL_i$ .

Proposition 2b. $VSL_i=\frac{S_i^U}{p_i^O(A_i)\beta^{A_i}u'(y_i^O(A_i))}$

This proposition follows from the definition of *VSL$_i$* as $\frac{\partial V_i/\partial p_i(A_i)}{\partial V_i/\partial y_i(A_i)}$, with these partial derivatives evaluated at *i*'s status quo risk and income profile (Definition 4). $\partial V_i/\partial p_i(A_i)$ evaluated at *i*'s status quo risk and income profile is just $S_i^U$. The alternative formula for $V_i$ (as demonstrated above) is:

$$V_i=\sum_{t=1}^{A_i-1}\beta^t u(y_i(t))+\sum_{t=A_i}^{T}\pi_i(t;A_i)\beta^t u(y_i(t))=\sum_{t=1}^{A_i-1}\beta^t u(y_i(t))+p_i(A_i)\beta^{A_i}u(y_i(A_i))+\sum_{t=A_i+1}^{T}\pi_i(t;A_i)\beta^t u(y_i(t))$$

. It follows that $\partial V_i/\partial y_i(A_i)$ evaluated at *i*'s status quo risk and income profile is: $p_i^O(A_i)\beta^{A_i}u'(y_i^O(A_i))$.

**Note regarding symbolism.** The remaining propositions and other results proved in this Appendix are manipulations of the formulas for SVRR$_i$ and VSL$_i$. Thus, in the remainder of the Appendix, as in the corresponding sections of the main text (Sections 3 and 4), we remove the "*O*" superscript on incomes, probabilities, and utilities, which is implicit. $y_i(t)$ denotes $y_i^O(t)$, $p_i(t)$ denotes $p_i^O(t)$, $V_i$ denotes $V_i^O$, and so forth. Further, we may drop subscripts on incomes or probabilities where two individuals *i* and *j* are being compared and these quantities are the same for *i* and *j*. For example, if $y_i(t)=y_j(t)$, then $y(t)$ may be used to denote $y_i(t)=y_j(t)$.

## II. Results in Section 3 of the Article

In all the propositions and other mathematical claims proved here, from Section 3 of the Article, *i* and *j* are two individuals with identical risk and income profiles, and *i* is older than *j* ($A_i > A_j$).

Section 3.1: Formula for $S_j^U - S_i^U$.

$$S_j^U - S_i^U = \sum_{t=A_j}^{A_i-1} \pi(t; A_j+1)\beta^t u(y(t)) + \left(\pi(A_i; A_j+1) - 1\right) \sum_{t=A_i}^{T} \pi(t; A_i+1)\beta^t u(y(t))$$

Using the formula derived above for $V_i$, $V_i = \sum_{t=1}^{A_i-1} \beta^t u(y_i(t)) + \sum_{t=A_i}^{T} \pi_i(t; A_i)\beta^t u(y_i(t))$ for a generic individual *i*, together with the definition of $S_i^U$ as the partial derivative of $W^U(\cdot)$ with respect to *i*'s current survival probability, we arrive at an alternative expression for $S_i^U$:

$$S_i^U = \sum_{t=A_i}^{T} \frac{\pi_i(t; A_i)}{p_i(A_i)} \beta^t u(y_i(t)).$$

Consider now the case at hand: *i* and *j* are two individuals with the same risk and income profiles, and $A_i > A_j$. $S_j^U - S_i^U = \sum_{t=A_j}^{A_i-1} \frac{\pi(t; A_j)}{p(A_j)} \beta^t u(y(t)) + \sum_{t=A_i}^{T} \left( \frac{\pi(t; A_j)}{p(A_j)} - \frac{\pi(t; A_i)}{p(A_i)} \right) \beta^t u(y(t))$. The first term on the RHS is $\sum_{t=A_j}^{A_i-1} \pi(t; A_j+1)\beta^t u(y(t))$, while the second term is equal to

$$\sum_{t=A_i}^{T} \left( \prod_{s=A_j+1}^{t} p(s) - \prod_{s=A_i+1}^{t} p(s) \right) \beta^t u(y(t)) = \sum_{t=A_i}^{T} \left( \pi(A_i; A_j+1)\pi(t; A_i+1) - \pi(t; A_i+1) \right) \beta^t u(y(t)) =$$
$$\left(\pi(A_i; A_j+1) - 1\right) \sum_{t=A_i}^{T} \pi(t; A_i+1)\beta^t u(y(t)) .$$

Section 3.1: If income is non-increasing with age and survival probabilities are non-increasing with age, the utilitarian SVRR decreases with age.

(a) Consider first the case in which $A_i \le T - 1$.

$$S_i^U = \sum_{t=A_i}^{T} \frac{\pi(t; A_i)}{p(A_i)} \beta^t u(y(t)) = \beta^{A_i} u(y(A_i)) + \sum_{t=A_i+1}^{T} \left( \prod_{s=A_i+1}^{t} p(s) \right) \beta^t u(y(t)).$$

$S_j^U = \beta^{A_j} u(y(A_j)) + \sum_{t=A_j+1}^{T-(A_i-A_j)} \left( \prod_{s=A_j+1}^{t} p(s) \right) \beta^t u(y(t)) + \sum_{t=T-(A_i-A_j)+1}^{T} \left( \prod_{s=A_j+1}^{t} p(s) \right) \beta^t u(y(t))$. Note that

the summations $\sum_{t=A_i+1}^{T}\left(\prod_{s=A_i+1}^{t} p(s)\right)\beta^t u(y(t))$ and $\sum_{t=A_j+1}^{T-(A_i-A_j)}\left(\prod_{s=A_j+1}^{t} p(s)\right)\beta^t u(y(t))$ each have ($T-A_i$) terms.

$$\sum_{t=A_j+1}^{T-(A_i-A_j)}\left(\prod_{s=A_j+1}^{t} p(s)\right)\beta^t u(y(t)) - \sum_{t=A_i+1}^{T}\left(\prod_{s=A_i+1}^{t} p(s)\right)\beta^t u(y(t))$$

$$= \sum_{t=A_j+1}^{T-(A_i-A_j)}\left[\left(\prod_{s=A_j+1}^{t} p(s)\right)\beta^t u(y(t)) - \left(\prod_{s=A_i+1}^{t+(A_i-A_j)} p(s)\right)\beta^{t+(A_i-A_j)} u(y(t+(A_i-A_j)))\right]$$

If income and survival probability are non-increasing with time, each term in the summation immediately above is non-negative.[2] It follows that $\sum_{t=A_j+1}^{T-(A_i-A_j)}\left(\prod_{s=A_j+1}^{t} p(s)\right)\beta^t u(y(t)) \geq \sum_{t=A_i+1}^{T}\left(\prod_{s=A_i+1}^{t} p(s)\right)\beta^t u(y(t))$. Moreover, if income is non-increasing with time, $\beta^{A_j} u(y(A_j)) \geq \beta^{A_i} u(y(A_i))$.[3] Finally, $\sum_{t=T-(A_i-A_j)+1}^{T}\left(\prod_{s=A_j+1}^{t} p(s)\right)\beta^t u(y(t)) > 0$. Thus $S_j^U > S_i^U$.

(b) Consider next the case in which $A_i = T$. In this case, $S_i^U = \beta^{A_i} u(y(A_i))$. $S_j^U = \beta^{A_j} u(y(A_j)) + \sum_{t=A_j+1}^{T}\left(\prod_{s=A_j+1}^{t} p(s)\right)\beta^t u(y(t))$. If income is non-decreasing with time, $\beta^{A_j} u(y(A_j)) \geq \beta^{A_i} u(y(A_i))$. $\sum_{t=A_j+1}^{T}\left(\prod_{s=A_j+1}^{t} p(s)\right)\beta^t u(y(t)) > 0$. Thus $S_j^U > S_i^U$.

Proposition 3a. The ex ante prioritarian SVRR displays Priority for the Young

As noted in the text, $S_j^{EAP} - S_i^{EAP} = g'(V_j)\left(S_j^U - S_i^U\right) + S_i^U\left(g'(V_j) - g'(V_i)\right)$. $V_j < V_i$ and thus $g'(V_j) > g'(V_i)$ by the strict concavity of $g(\cdot)$. $S_i^U > 0$, and thus $S_i^U\left(g'(V_j) - g'(V_i)\right) > 0$. Finally, $g'(V_j) > 0$ by the strict increasingness of $g(\cdot)$. It therefore follows that if $S_j^U - S_i^U > 0$, then $S_j^{EAP} - S_i^{EAP} > 0$; and if $S_j^U - S_i^U = 0$, then $S_j^{EAP} - S_i^{EAP} > 0$.

[2] If income and survival probabilities are constant and β = 1, each term is 0 rather than positive.

[3] If income is constant and β = 1, these two quantities are equal.

Proposition 3b. The ex ante prioritarian SVRR displays Ratio Priority for the Young

$(S_j^{EAP}/S_i^{EAP}) = \frac{g'(V_j)}{g'(V_i)}\frac{S_j^U}{S_i^U}$. Because $g(\cdot)$ is strictly increasing and strictly concave and $V_j$ < $V_i$, we have that $\frac{g'(V_j)}{g'(V_i)} > 1$. Because $S_j^U > 0$ and $S_i^U > 0$, $\frac{S_j^U}{S_i^U} > 0$ and hence $\frac{g'(V_j)}{g'(V_i)}\frac{S_j^U}{S_i^U} > \frac{S_j^U}{S_i^U}$.

Section 3.3: Formula for $S_j^{EPP} - S_i^{EPP}$

$$S_j^{EPP} - S_i^{EPP} = \sum_{t=A_j}^{A_i-1}\mu(t;A_j+1)g(U(t)) + (\pi(A_i;A_j+1)-1)\sum_{t=A_i}^{T}\mu(t;A_i+1)g(U(t)) + \left(g(U(A_i-1)) - g(U(A_j-1))\right)$$

, with $i$ and $j$ two individuals with the same risk and income profiles¸ and $A_i > A_j$.

Using the formula for the ex post prioritarian SVRR (Proposition 1c), we have that

$$S_j^{EPP} - S_i^{EPP} = \sum_{t=A_j}^{A_i-1}\frac{\mu(t;A_j)}{p(A_j)}g(U(t)) + \sum_{t=A_i}^{T}\left(\frac{\mu(t;A_j)}{p(A_j)} - \frac{\mu(t;A_i)}{p(A_i)}\right)g(U(t)) + \left(g(U(A_i-1)) - g(U(A_j-1))\right)$$

. The first term on the RHS is $\sum_{t=A_j}^{A_i-1}\mu(t;A_j+1)g(U(t))$. The second term is equal to:

$$\sum_{t=A_i}^{T}\left((1-p(t+1))\prod_{s=A_j+1}^{t}p(s) - (1-p(t+1))\prod_{s=A_i+1}^{t}p(s)\right)g(U(t)) = \sum_{t=A_i}^{T}\left(\pi(A_i;A_j+1)\mu(t;A_i+1) - \mu(t;A_i+1)\right)g(U(t))$$

$$= (\pi(A_i;A_j+1)-1)\sum_{t=A_i}^{T}\mu(t;A_i+1)g(U(t)).$$

Proposition 3c. The ex post prioritarian SVRR displays Priority for the Young.

Rather than prove this directly, we prove Proposition 3d, below, namely that the ex post prioritarian SVRR displays Ratio Priority for the Young. Ratio Priority for the Young is logically stronger than Priority for the Young (see Article, note 31), i.e., Proposition 3c follows from Proposition 3d.

Proposition 3d. The ex post prioritarian SVRR displays Ratio Priority for the Young.

In what follows, we'll abbreviate $g(U(A_j-1))$ and $g(U(A_i-1))$ as $g_{(A_j-1)}$ and $g_{(A_i-1)}$, respectively; and $U(A_j-1)$ and $U(A_i-1)$ as $U_{(A_j-1)}$ and $U_{(A_i-1)}$, respectively. $\pi(A_i)$ and $\pi(A_j)$ are shorthand for $\pi(A_i;1)$ and $\pi(A_j;1)$, respectively.

$S_j^{EPP} = -g_{(A_j-1)} + \sum_{t=A_j}^{A_i-1} \frac{\mu(t;A_j)}{p(A_j)} g(U(t)) + \sum_{t=A_i}^{T} \frac{\mu(t;A_j)}{p(A_j)} g(U(t))$. Note now that

$\sum_{t=A_i}^{T} \frac{\mu(t;A_j)}{p(A_j)} = \frac{\pi(A_i)}{\pi(A_j)}$ and $\sum_{t=A_j}^{A_i-1} \frac{\mu(t;A_j)}{p(A_j)} = 1 - \frac{\pi(A_i)}{\pi(A_j)}$. Thus we have that

$$S_j^{EPP} = \frac{\pi(A_i)}{\pi(A_j)}(g_{(A_i-1)} - g_{(A_j-1)}) + \sum_{t=A_j}^{A_i-1} \frac{\mu(t;A_j)}{p(A_j)}\left(g(U(t)) - g_{(A_j-1)}\right) + \sum_{t=A_i}^{T} \frac{\mu(t;A_j)}{p(A_j)}\left(g(U(t)) - g_{(A_i-1)}\right).$$

$$S_i^{EPP} = -g_{(A_i-1)} + \sum_{t=A_i}^{T} \frac{\mu(t;A_i)}{p(A_i)} g(U(t)) = \frac{\pi(A_j)}{\pi(A_i)} \sum_{t=A_i}^{T} \frac{\mu(t;A_j)}{p(A_j)}\left(g(U(t)) - g_{(A_i-1)}\right) .$$

Turning to the utilitarian SVRR: we can proceed by steps parallel to those immediately above to derive the following expressions for $S_i^U$ and $S_j^U$.

$$S_j^U = \frac{\pi(A_i)}{\pi(A_j)}(U_{(A_i-1)} - U_{(A_j-1)}) + \sum_{t=A_j}^{A_i-1} \frac{\mu(t;A_j)}{p(A_j)}\left(U(t) - U_{(A_j-1)}\right) + \sum_{t=A_i}^{T} \frac{\mu(t;A_j)}{p(A_j)}\left(U(t) - U_{(A_i-1)}\right) .$$

$$S_i^U = \frac{\pi(A_j)}{\pi(A_i)} \sum_{t=A_i}^{T} \frac{\mu(t;A_j)}{p(A_j)}\left(U(t) - U_{(A_i-1)}\right).$$

Observe that $\frac{S_j^{EPP}}{S_i^{EPP}} = \frac{\frac{\pi(A_i)}{\pi(A_j)}(g_{(A_i-1)} - g_{(A_j-1)}) + \sum_{t=A_j}^{A_i-1} \frac{\mu(t;A_j)}{p(A_j)}\left(g(U(t)) - g_{(A_j-1)}\right)}{\frac{\pi(A_j)}{\pi(A_i)} \sum_{t=A_i}^{T} \frac{\mu(t;A_j)}{p(A_j)}\left(g(U(t)) - g_{(A_i-1)}\right)} + \frac{\pi(A_i)}{\pi(A_j)}$, and

that $\frac{S_j^U}{S_i^U} = \frac{\frac{\pi(A_i)}{\pi(A_j)}(U_{(A_i-1)} - U_{(A_j-1)}) + \sum_{t=A_j}^{A_i-1} \frac{\mu(t;A_j)}{p(A_j)}\left(U(t) - U_{(A_j-1)}\right)}{\frac{\pi(A_j)}{\pi(A_i)} \sum_{t=A_i}^{T} \frac{\mu(t;A_j)}{p(A_j)}\left(U(t) - U_{(A_i-1)}\right)} + \frac{\pi(A_i)}{\pi(A_j)}$. Thus $\frac{S_j^{EPP}}{S_i^{EPP}} > \frac{S_j^U}{S_i^U}$ iff

$$\frac{\frac{\pi(A_i)}{\pi(A_j)}(g_{(A_i-1)} - g_{(A_j-1)}) + \sum_{t=A_j}^{A_i-1} \frac{\mu(t;A_j)}{p(A_j)}\left(g(U(t)) - g_{(A_j-1)}\right)}{\frac{\pi(A_j)}{\pi(A_i)} \sum_{t=A_i}^{T} \frac{\mu(t;A_j)}{p(A_j)}\left(g(U(t)) - g_{(A_i-1)}\right)} > \frac{\frac{\pi(A_i)}{\pi(A_j)}(U_{(A_i-1)} - U_{(A_j-1)}) + \sum_{t=A_j}^{A_i-1} \frac{\mu(t;A_j)}{p(A_j)}\left(U(t) - U_{(A_j-1)}\right)}{\frac{\pi(A_j)}{\pi(A_i)} \sum_{t=A_i}^{T} \frac{\mu(t;A_j)}{p(A_j)}\left(U(t) - U_{(A_i-1)}\right)}$$

Equivalently, $\frac{S_j^{EPP}}{S_i^{EPP}} > \frac{S_j^U}{S_i^U}$ iff

$$\frac{\frac{\pi(A_i)}{\pi(A_j)}\left(g_{(A_i-1)} - g_{(A_j-1)}\right) + \sum_{t=A_j}^{A_i-1}\frac{\mu(t;A_j)}{p(A_j)}\left(g(U(t)) - g_{(A_j-1)}\right)}{\frac{\pi(A_i)}{\pi(A_j)}\left(U_{(A_i-1)} - U_{(A_j-1)}\right) + \sum_{t=A_j}^{A_i-1}\frac{\mu(t;A_j)}{p(A_j)}\left(U(t) - U_{(A_j-1)}\right)} > \frac{\sum_{t=A_i}^{T}\frac{\mu(t;A_j)}{p(A_j)}\left(g(U(t)) - g_{(A_i-1)}\right)}{\sum_{t=A_i}^{T}\frac{\mu(t;A_j)}{p(A_j)}\left(U(t) - U_{(A_i-1)}\right)} .$$

Let $\theta = \frac{g_{(A_i)} - g_{(A_i-1)}}{U_{(A_i)} - U_{(A_i-1)}}$ . Note that $\frac{\sum_{t=A_i}^{T}\frac{\mu(t;A_j)}{p(A_j)}\left(g(U(t)) - g_{(A_i-1)}\right)}{\sum_{t=A_i}^{T}\frac{\mu(t;A_j)}{p(A_j)}\left(U(t) - U_{(A_i-1)}\right)} \leq \theta$. This is because—by the strict concavity of $g(\cdot)$—each term in the numerator of the preceding fraction is less than or equal to θ times the corresponding term in the denominator. Similarly,

$$\frac{\frac{\pi(A_i)}{\pi(A_j)}\left(g_{(A_i-1)} - g_{(A_j-1)}\right) + \sum_{t=A_j}^{A_i-1}\frac{\mu(t;A_j)}{p(A_j)}\left(g(U(t)) - g_{(A_j-1)}\right)}{\frac{\pi(A_i)}{\pi(A_j)}\left(U_{(A_i-1)} - U_{(A_j-1)}\right) + \sum_{t=A_j}^{A_i-1}\frac{\mu(t;A_j)}{p(A_j)}\left(U(t) - U_{(A_j-1)}\right)} > \theta .$$

<u>Proposition 3e</u>. VSL does not display Priority for the Young.

This proposition is proved in the text of the Article.

<u>Proposition 3f</u>. VSL does not display Ratio Priority for the Young.

As noted in the Article, this proposition follows from Proposition 3e because Ratio Priority for the Young is logically stronger than Priority for the Young. It can also easily be proved directly. Let $C_i = p(A_i)\beta^{A_i}u'(y(A_i))$ and similarly for $C_j$. $\frac{VSL_j}{VSL_i} = \frac{C_i}{C_j}\frac{S_j^U}{S_i^U}$ . $\frac{S_j^U}{S_i^U} > 0$ since $S_j^U$ , $S_i^U$ > 0. $C_i > 0$, $C_j > 0$. It's possible that $C_i \leq C_j$, e.g., if $p(A_i) \leq p(A_j)$ and $u'(y(A_i)) \leq u'(y(A_j))$, and indeed that $C_i < C_j$. So it's possible that $\frac{C_i}{C_j}\frac{S_j^U}{S_i^U} \leq \frac{S_j^U}{S_i^U}$ and indeed that $\frac{C_i}{C_j}\frac{S_j^U}{S_i^U} < \frac{S_j^U}{S_i^U}$ .

## III. Results in Section 4.1 of the Article

For purposes of proving propositions 4a, 4b, 4c, and 4d, we assume that *i* and *j* are identical in age ($A_i = A_j$), in their risk profiles, and in their income profiles except that $y_j(t) = y_i(t) + \Delta y$, $\Delta y > 0$, for some single period *t*—a past period, the current period ($t = A_i = A_j$) or a future period. We determine whether $\text{SVRR}_j > \text{SVRR}_i$, $\text{SVRR}_j = \text{SVRR}_i$, or $\text{SVRR}_j < \text{SVRR}_i$ by examining the sign of $\frac{\partial S_i}{\partial y_i(t)}$. We proceed analogously for VSL. Statements regarding increments to future periods assume that $A_i = A_j \le T - 1$.

Proposition 4a. The utilitarian SVRR is unchanged by a single-period increment to past income. It increases with a single-period increment to present or future income.

As shown above (see Appendix, p. 6), $S_i^U = \sum_{t=A_i}^{T} \frac{\pi_i(t;A_i)}{p_i(A_i)} \beta^t u(y_i(t))$. $\frac{\partial S_i^U}{\partial y_i(t)} = 0$ for $t < A_i$.

$\frac{\partial S_i^U}{\partial y_i(t)} = \frac{\pi_i(t;A_i)}{p_i(A_i)} \beta^t u'(y_i(t)) > 0$ for all $t \ge A_i$.

Proposition 4b. The ex ante prioritarian SVRR decreases with a single-period increment to past income. The effect of a single-period increment to present income or future income on the ex ante prioritarian SVRR is ambiguous.

For $t < A_i$, $\frac{\partial S_i^{EAP}}{\partial y_i(t)} = g''(V_i) S_i^U \beta^t u'(y_i(t)) < 0$.

For $t \ge A_i$, $\frac{\partial S_i^{EAP}}{\partial y_i(t)} = \frac{\pi_i(t;A_i)}{p_i(A_i)} \beta^t u'(y_i(t)) \left( g''(V_i) \sum_{s=A_i}^{T} \pi_i(s;A_i) \beta^s u(y_i(s)) + g'(V_i) \right)$. This expression is positive/negative/zero iff $-\frac{g''(V_i)}{g'(V_i)}$ is less than/greater than/equal to

$$\frac{1}{\sum_{s=A_i}^{T} \pi_i(s;A_i) \beta^s u(y_i(s))}.$$

Note that $V_i > \sum_{s=A_i}^{T} \pi_i(s;A_i) \beta^s u(y_i(s))$. Thus, manipulating the above equation, we have the following: if $-\frac{g''(V_i)V_i}{g'(V_i)} \le 1$, then $\frac{\partial S_i^{EAP}}{\partial y_i(t)} > 0$. In short, if $g(\cdot)$ is such that the coefficient of relative risk aversion is always less than or equal to 1, a one-time increment to present or future income will increase the ex ante prioritarian SVRR.

Proposition 4c. The ex post prioritarian SVRR decreases with a single-period increment to past income. It increases with a single-period increment to present or future income.

For $t < A_i$, $\dfrac{\partial S_i^{EPP}}{\partial y_i(t)} = -\beta^t u'(y_i(t))\left[ g'(U_i(A_i - 1)) - \sum_{s=A_i}^{T} \dfrac{\mu_i(s;A_i)}{p_i(A_i)} g'(U_i(s)) \right] < 0$.

For $t \geq A_i$, $\dfrac{\partial S_i^{EPP}}{\partial y_i(t)} = \beta^t u'(y_i(t)) \sum_{s=t}^{T} \dfrac{\mu_i(s;A_i)}{p_i(A_i)} g'(U_i(s)) > 0$.

Proposition 4d. VSL is unchanged by a single-period increment to past income. It increases with a single-period increment to present or future income.

Let $C_i = p_i(A_i)\beta^{A_i} u'(y_i(A_i))$. $VSL_i = \dfrac{S_i^U}{C_i}$ , $C_i > 0$.

For $t < A_i$, $t > A_i$, $\dfrac{\partial VSL_i}{\partial y_i(t)} = \dfrac{1}{C_i}\dfrac{\partial S_i^U}{\partial y_i(t)}$. $\dfrac{\partial S_i^U}{\partial y_i(t)} = 0$ for $t < A_i$ and $\dfrac{\partial S_i^U}{\partial y_i(t)} > 0$ for $t > A_i$ (see above, Proposition 4a), hence the same is true of $\dfrac{\partial VSL_i}{\partial y_i(t)}$.

For $t = A_i$, $\dfrac{\partial VSL_i}{\partial y_i(t)} = \left(\dfrac{1}{C_i}\right)^2 \left( C_i \dfrac{\partial S_i^U}{\partial y_i(t)} - S_i^U p_i(A_i)\beta^{A_i} u''(y_i(A_i)) \right)$. $\dfrac{\partial S_i^U}{\partial y_i(t)} > 0$ (see Proposition 4a); and $S_i^U p_i(A_i)\beta^{A_i} u''(y_i(A_i)) < 0$. Hence $\dfrac{\partial VSL_i}{\partial y_i(t)} > 0$.

Propositions 5a, 5b, 5c and 5d concern the effect on SVRR and VSL of an increment to permanent income. Here, we assume that two individuals $i$ and $j$ are identical except that $y_j(t) = y_i(t) + \Delta y$, $\Delta y > 0$, for all periods.

Proposition 5a. The utilitarian SVRR increases with an increment to permanent income.

Because the utilitarian SVRR is unchanged by a single-period increment to past income, and increases with a single-period increment to present or future income, it clearly increases with an increment to permanent income.

Proposition 5b. The effect of an increment to permanent income on the ex ante prioritarian SVRR is ambiguous.

An example suffices to prove that the effect of an increment to permanent income is ambiguous. Let $T=2$, $\beta = 1$, $A_i = A_j = 2$, $p_i(2) = p$, and $y_i(1) = y_i(2) = y$. Then $V_i = u(y)(1 + p)$. Denote $(1 + p)$ as $q$. $S_i^{EAP} = (u(y))g'(u(y)q)$. $\frac{\partial S_i^{EAP}}{\partial y} = u'(y)\left(g''(u(y)q)u(y)q + g'(u(y)q)\right)$. The sign of this expression is positive iff $g''(u(y)q)u(y)q + g'(u(y)q) > 0$ or, equivalently, $-\frac{g''(u(y)q)}{g'(u(y)q)}u(y)q < 1$.

<u>Proposition 5c</u>. The effect of an increment to permanent income on the ex post prioritarian SVRR is ambiguous.

An example suffices to prove that the effect of an increment to permanent income is ambiguous. Let $T=2$, $\beta = 1$, $A_i = A_j = 2$, $p_i(2) = p$, and $y_i(1) = y_i(2) = y$. Then $S_i^{EPP} = g(2u(y)) - g(u(y))$. $\frac{\partial S_i^{EPP}}{\partial y} = 2g'(2u(y))u'(y) - g'(u(y))u'(y)$, the sign of which depends upon $2g'(2u(y)) - g'(u(y))$. Depending on $g(\cdot)$, this term can be positive, negative, or zero for $u(y) > 0$.

<u>Proposition 5d</u>. VSL increases with an increment to permanent income.

Because VSL is unchanged by a single-period increment to past income, and increases with a single-period increment to present or future income, it clearly increases with an increment to permanent income.

## IV. Results in Section 4.2 of the Article

For purposes of proving Propositions 6a, 6b, 6c, and 6d, we assume that $i$ and $j$ are identical in age ($A_i = A_j$), in their income profiles, and in their risk profiles except that $p_j(t) = p_i(t) + \Delta q$, $\Delta q > 0$, for some single period $t$—either the current period ($t = A_i = A_j$) or a future period. We determine whether $\text{SVRR}_j > \text{SVRR}_i$, $\text{SVRR}_j = \text{SVRR}_i$, or $\text{SVRR}_j < \text{SVRR}_i$ by examining the sign of $\frac{\partial S_i}{\partial p_i(t)}$. We proceed analogously for VSL. Statements regarding increments to future periods assume that $A_i = A_j \leq T - 1$.

<u>Proposition 6a</u>. The utilitarian SVRR is unchanged by a single-period increment to present survival probability. It increases with a single-period increment to future survival probability.

$$S_i^U = \sum_{t=A_i}^{T} \frac{\pi_i\,(t;A_i)}{p_i\,(A_i)} \beta^t u(y_i\,(t))$$. (See above, Appendix p. 6)

$\sum_{t=A_i}^{T} \frac{\pi_i\,(t;A_i)}{p_i\,(A_i)} \beta^t u(y_i\,(t)) = \beta^{A_i} u(y_i(A_i)) + \sum_{t=A_i+1}^{T} \beta^t u(y_i(t)) \prod_{s=A_i+1}^{t} p_i(s)$. Hence $p_i(A_i)$ is not an argument of $S_i^U$, and $\frac{\partial S_i^U}{\partial p_i(t)} = 0$ for $t = A_i$.

For $t > A_i$, $\frac{\partial S_i^U}{\partial p_i(t)} = \sum_{s=t}^{T} \frac{\pi_i(s;A_i)}{p_i(A_i)} \beta^s u(y_i(s)) \frac{1}{p_i(t)} > 0$.

<u>Proposition 6b</u>. The ex ante prioritarian SVRR decreases with a single-period increment to present survival probability. The effect of a single-period increment to future survival probability on the ex ante prioritarian SVRR is ambiguous.

For $t = A_i$, $\frac{\partial S_i^{EAP}}{\partial p_i(t)} = g''(V_i)\left(S_i^U\right)^2$. To see this, note that $S_i^U = \frac{\partial V_i}{\partial p_i(t)}$ for $t = A_i$; and that $\frac{\partial S_i^U}{\partial p_i(t)} = 0$ for $t = A_i$, by Proposition 6a. $g''(V_i)\left(S_i^U\right)^2 < 0$.

For $t > A_i$, $\frac{\partial S_i^{EAP}}{\partial p_i(t)} = \sum_{s=t}^{T} \frac{\pi_i(s;A_i)}{p_i(A_i)} \beta^s u(y_i(s)) \frac{1}{p_i(t)} \left[ g'(V_i) + g''(V_i) \sum_{s=A_i}^{T} \pi_i(s;A_i) \beta^s u(y_i(s)) \right]$.

This is greater than/equal to/less than 0 iff $\frac{-g''(V_i)}{g'(V_i)}$ is less than/equal to/greater than $\frac{1}{\sum_{s=A_i}^{T} \pi_i(s;A_i)\beta^s u(y_i(s))}$. Observing again that $V_i > \sum_{s=A_i}^{T} \pi_i(s;A_i)\beta^s u(y_i(s))$, we have a parallel result here as for the effect of present income and future income (see above, demonstration of Proposition 4b): a one-period increment to future survival probability will increase the ex ante prioritarian SVRR if the coefficient of relative risk aversion for $g(\cdot)$ is uniformly less than or equal to one.

<u>Proposition 6c</u>. The ex post prioritarian SVRR is unchanged by a single-period increment to present survival probability. It increases with a single-period increment to future survival probability.

$$S_i^{EPP} = -g(U_i\,(A_i - 1)) + \sum_{t=A_i}^{T} \frac{\mu_i\,(t;A_i)}{p_i\,(A_i)} g(U_i(t))$$

$$= -g(U_i\,(A_i - 1)) + g(U_i(A_i))(1 - p_i(A_i + 1)) + \sum_{t=A_i+1}^{T} \left( \prod_{s=A_i+1}^{t} p_i(s) \right)(1 - p_i(t+1))g(U_i(t))\ .$$

Hence $p_i(A_i)$ is not an argument of $S_i^{EPP}$ , and $\dfrac{\partial S_i^{EPP}}{\partial p_i(t)} = 0$ for $t = A_i$.

For $t > A_i$, $\dfrac{\partial S_i^{EPP}}{\partial p_i(t)} = -\dfrac{\pi_i(t-1; A_i)}{p_i(A_i)} g(U_i(t-1)) + \sum_{s=t}^{T} \dfrac{\mu_i(s; A_i)}{p_i(A_i)\,p_i(t)} g(U_i(s))$. Note that $\pi_i(t-1; A_i) = \sum_{s=t}^{T} \dfrac{\mu_i(s; A_i)}{p_i(t)}$, and that $g(U_i(s)) > g(U_i(t-1))$ for $s \geq t$ by the strict increasingness of $g(\cdot)$. Hence, for $t > A_i$,

$$-\frac{\pi_i(t-1; A_i)}{p_i(A_i)} g(U_i(t-1)) + \sum_{s=t}^{T} \frac{\mu_i(s; A_i)}{p_i(A_i)\,p_i(t)} g(U_i(s)) > -\frac{\pi_i(t-1; A_i)}{p_i(A_i)} g(U_i(t-1)) + \sum_{s=t}^{T} \frac{\mu_i(s; A_i)}{p_i(A_i)\,p_i(t)} g(U_i(t-1)) = 0$$

Proposition 6d. VSL decreases with a single-period increment to present survival probability. It increases with a single-period increment to future survival probability.

Let $C_i = p_i(A_i)\beta^{A_i} u'(y_i(A_i))$. $VSL_i = \dfrac{S_i^U}{C_i}$ , $C_i > 0$. For $t = A_i$,

$\dfrac{\partial VSL_i}{\partial p_i(t)} = \dfrac{-S_i^U \beta^{A_i} u'(y_i(A_i))}{(C_i)^2} < 0$. (Recall that $\dfrac{\partial S_i^U}{\partial p_i(t)} = 0$ for $t = A_i$; see Proposition 6a.)

For $t > A_i$, $\dfrac{\partial VSL_i}{\partial p_i(t)} = \dfrac{1}{(C_i)} \dfrac{\partial S_i^U}{\partial p_i(t_i)} > 0$ because $\dfrac{S_i^U}{\partial p_i(t)} > 0$ (Proposition 6a).

Propositions 7a, 7b, 7c and 7d concern the effect on SVRR and VSL of a permanent increment to (present and future) survival probability. Here, we assume that two individuals $i$ and $j$ are identical except that $p_j(t) = p_i(t) + \Delta q$, $\Delta q > 0$, for all present and future periods.

Proposition 7a. The utilitarian SVRR increases with a permanent increment to survival probability.

Because the utilitarian SVRR is unchanged by a single-period increment to present survival probability, and increases with a single-period increment to future survival probability, it clearly increases with a permanent increment to survival probability.

Proposition 7b. The effect of a permanent increment to survival probability on the ex ante prioritarian SVRR is ambiguous.

An example is sufficient to prove that the impact of a permanent increment in survival probability is ambiguous. Let $T = 3$, $\beta = 1$, $A_i = 2$, $p_i(2) = p_i(3) = p$, and $y_i(1) = y_i(2) = y_i(3) = y$. $V_i = u(y)[1 + p + p^2]$, and $S_i^{EAP} = g'(V_i)u(y)(1+p)$.

$\frac{\partial S_i^{EAP}}{\partial p} = g'(V_i)u(y) + u(y)(1+p)g''(V_i)u(y)(1+2p)$. Note, in turn, that

$g'(V_i)u(y) + u(y)(1+p)g''(V_i)u(y)(1+2p) >< 0$ iff $\frac{-g''(V_i)V_i}{g'(V_i)} <> \frac{1+p+p^2}{1+3p+2p^2}$. This last term is bounded below by ½ and above by 1. Thus the ex ante prioritarian SVRR in this case increases with a permanent increment in survival probability if the coefficient of relative risk aversion is sufficiently small, and decreases with a permanent increment in survival probability if the coefficient of relative risk aversion is sufficiently large.

Proposition 7c: The ex post prioritarian SVRR increases with a permanent increment to survival probability.

Because the ex post prioritarian SVRR is unchanged by a single-period increment to present survival probability, and increases with a single-period increment to future survival probability, it clearly increases with a permanent increment to survival probability.

Proposition 7d. The effect of a permanent increment to survival probability on VSL is ambiguous.

An example is sufficient to prove that the impact of a permanent increment to survival probability is ambiguous. Let $T = 5$, $\beta = 1$, $A_i = 2$; $p_i(t) = p$ for $t \geq 2$; $y_i(t) = y$ for all $t$.
$S_i^U = (1+p+p^2+p^3)u(y)$, and $VSL_i = \frac{S_i^U}{pu'(y)}$. $\frac{\partial VSL_i}{\partial p} = \frac{(p^2+2p^3-1)u(y)u'(y)}{(pu'(y))^2}$. Since $u(y)$, $u'(y)$ and $p > 0$, we have that $\frac{\partial VSL_i}{\partial p} > 0$ if $p$ is sufficiently large that $p^2 + 2p^3 - 1 > 0$; and that $\frac{\partial VSL_i}{\partial p} < 0$ if $p^2 + 2p^3 - 1 < 0$.
